# UV light and adaptive divergence of leaf physiology, anatomy, and ultrastructure drive heat stress tolerance in genetically distant grapevines

**DOI:** 10.3389/fpls.2024.1399840

**Published:** 2024-06-18

**Authors:** Ana Fernandes de Oliveira, Giovanni Kamal Piga, Soumiya Najoui, Giovanna Becca, Salvatore Marceddu, Maria Pia Rigoldi, Daniela Satta, Simonetta Bagella, Giovanni Nieddu

**Affiliations:** ^1^ Agris Sardegna, Agricultural Research Agency of Sardinia, Sassari, Italy; ^2^ Department of Agriculture, University of Sassari, Sassari, Italy; ^3^ Department of Chemical, Physical, Mathematical and Natural Sciences, University of Sassari, Sassari, Italy; ^4^ Institute of Sciences of Food Production, National Research Council, Sassari, Italy

**Keywords:** *Vitis vinifera* L., Carignano, Cannonau, stress tolerance, UV-B light, *in vivo* metabolite dynamics, leaf reflectance, leaf cell barriers

## Abstract

The genetic basis of plant response to light and heat stresses had been unveiled, and different molecular mechanisms of leaf cell homeostasis to keep high physiological performances were recognized in grapevine varieties. However, the ability to develop heat stress tolerance strategies must be further elucidated since the morpho-anatomical and physiological traits involved may vary with genotype × environment combination, stress intensity, and duration. A 3-year experiment was conducted on potted plants of Sardinian red grapevine cultivars Cannonau (syn. Grenache) and Carignano (syn. Carignan), exposed to prolonged heat stress inside a UV-blocking greenhouse, either submitted to low daily UV-B doses of 4.63 kJ m^−2^ d^−1^ (+UV) or to 0 kJ m^−2^ d^−1^ (−UV), and compared to a control (C) exposed to solar radiation (4.05 kJ m^−2^ d^−1^ average UV-B dose). Irrigation was supplied to avoid water stress, and canopy light and thermal microclimate were monitored continuously. Heat stress exceeded one-third of the duration inside the greenhouse and 6% in C. *In vivo* spectroscopy, including leaf reflectance and fluorescence, allowed for characterizing different patterns of leaf traits and metabolites involved in oxidative stress protection. Cannonau showed lower stomatal conductance under C (200 mmol m^−2^ s^−1^) but more than twice the values inside the greenhouse (400 to 900 mmol m^−2^ s^−1^), where water use efficiency was reduced similarly in both varieties. Under severe heat stress and −UV, Cannonau showed a sharper decrease in primary photochemical activity and higher leaf pigment reflectance indexes and leaf mass area. UV-B increased the leaf pigments, especially in Carignano, and different leaf cell regulatory traits to prevent oxidative damage were observed in leaf cross-sections. Heat stress induced chloroplast swelling, plastoglobule diffusion, and the accumulation of secretion deposits in both varieties, aggravated in Cannonau −UV by cell vacuolation, membrane dilation, and diffused leaf blade spot swelling. Conversely, in Carignano UV-B, cell wall barriers and calcium oxalate crystals proliferated in mesophyll cells. These responses suggest an adaptive divergence among cultivars to prolonged heat stress and UV-B light. Further research on grapevine biodiversity, heat, and UV-B light interactions may give new insights on the extent of stress tolerance to improve viticulture adaptive strategies in climate change hotspots.

## 1 Introduction

The ongoing global warming will keep impacting grapevine cultivation worldwide in the next decades, with prolonged heat and drought conditions in Mediterranean areas, higher frequency of frost, hail, and/or extreme heat waves during berry development (Droulia and Charalampopoulos, 2023; IPCC, 2023). In recent years, the scientific community devoted many efforts to develop knowledge concerning grapevine genetical, biochemical, and molecular responses to abiotic stresses in order to cope and define adaptation strategies to climate change in viticulture (Bernardo et al., 2018; Gomès et al., 2021; Pastore et al., 2022; Hewitt et al., 2023; Jiao et al., 2023). New farming strategies, including rootstocks and canopy, soil, and water management, have been proposed to optimize the use of natural resources and to address multiple stresses with more efficient cultural practices (Gutiérrez-Gamboa et al., 2021; Marín et al., 2021; Sosa-Zuniga et al., 2022; Yang et al., 2022; Poni et al., 2023), both from economic and ecological perspectives. These may allow to mitigate climate change-related events, like frequent frost and hail damages during flowering and fruit development stages (Mosedale et al., 2016) or prolonged heat and drought during berry development, that lead to yield reductions and berry quality losses (Sadras and Moran, 2012; Leolini et al., 2019; Rogiers et al., 2022; Fernández-Zurbano et al., 2024).

Regarding the genetic basis and phenotypical mechanisms activated in response to light, heat, and/or water stresses, many findings have been reported (Wahid et al., 2007; Salem-Fnayou et al., 2011; Janni et al., 2020; Ferrandino et al., 2023), and different molecular mechanisms to keep leaf cell homeostasis and high physiological performances were identified in grapevine varieties adapted to different environmental conditions (Pelsy, 2010; Carvalho et al., 2015a). The main changes in plant anatomy are common to that observed under drought and include cell size reduction, enlarged xylem vessels, increased leaf blade thickness, stomatal and trichoma density, and a smaller proportion of palisade mesophyll (Wahid et al., 2007; Salem-Fnayou et al., 2011). Plant physiological performance is reduced, with increased stomatal closure, reduced transpiration, and consequent reduced source–sink activity. Photosynthetic performance is affected also by chloroplast swelling and structural changes in thylakoid lamellae that lead to grana separation and to scattered or empty PSII antennae (Zahra et al., 2023). At the cellular level, gradual exposure to heat determines enzymatic inactivation in chloroplasts and mitochondria, inhibition of protein synthesis, and enhanced plasma membrane permeability, whereas direct severe damages caused by very high temperature are related to increased lipid fluidity in membranes and protein denaturation, hence the loss of membrane integrity (Haider et al., 2021). Grapevine responses and acclimation to heat stress, either caused by abrupt increases or gradual exposure to high temperatures, involve deep alteration in gene expression and transcript accumulation, coding for primary or secondary metabolism proteins and leading to the synthesis of different stress-related proteins that associate with cell wall, chloroplasts, ribosomes, and mitochondria, preserving their structural integrity and function and preventing other protein denaturation (Wahid et al., 2007; Ferrandino et al., 2023). Different transcription factors and heat shock proteins may be involved in abiotic stress responses, therefore establishing different stress tolerance strategies (Janni et al., 2020). Simultaneously, the complex hormonal signaling network (namely, abscisic acid, jasmonate, or carotenoid-derived hormone crosstalk pathways) triggers the activation of defense mechanisms, including enzymatic and non-enzymatic detoxification of reactive oxygen species to protect cells from oxidative injuries, including the synthesis of secondary metabolites such as flavonoids, glutathione, and ascorbic acid (Chen et al., 2022; Ferrandino et al., 2023). Under diverse genotype–environmental stress combinations (e.g., heat, drought, and UV-B light), the expression of genotype-specific heat shock proteins (HSP) determine cell and tissue stress tolerance strategy and the accumulation of different compatible solutes like sugar, sugar-alcohols, proline, and other osmolytes that buffer cellular redox potential and mediate flavonoid biosynthesis. Therefore, genotype-specific HSP allow for the improvement of membrane stability, thus reducing the impact of high temperatures on leaf gas exchanges and water use efficiency as well as on nutrient and photosynthate partitioning and plant growth (Wahid et al., 2007; Ferrandino et al., 2023). Plant exposure to high UV-B doses may lead to the excessive production of ROS and reduced photosynthesis, impairing photochemical efficiency, reducing photosynthetic pigment accumulation, and inducing the proteolytic degradation of Rubisco. On the contrary, low doses of UV-B radiation positively influence antioxidant defenses, being able to activate the photomorphogenic signaling of flavonoid biosynthesis and regulation of ROS homeostasis (Das and Roychoudhury, 2014). Besides the duration and doses of UV-B, the extent of antioxidant mechanism activation may vary among plant genotypes (Tan et al., 2023).

Among the adaptive strategies employed to keep optimal quality standards in grape-growing regions that are now facing systematic prolonged drought and heat stresses, vineyard cultivation in high altitudes can benefit vine physiology and berry metabolism and wine quality (Mercenaro et al., 2019; Arias et al., 2022), especially due to the lower duration of elevated temperatures and to the positive effects of UV-B radiation on vine physiology (Martínez-Lüscher et al., 2015) and anthocyanin and phenolic metabolism (Martínez-Lüscher et al., 2014; 2016a; Fernandes de Oliveira and Nieddu, 2016a). The important role played by local biodiversity on vineyard plasticity in a changing climate must be further considered when evaluating the best viticulture strategies to cope with global warming since not only the local varieties have justified grapevine cultivation for centuries but they also guaranteed acclimation under local environments (Merrill et al., 2020; Antolín et al., 2021; Naulleau et al., 2021). Therefore, other than the different plant agronomical traits and berry characteristics that determined vineyard resilience and wine typicity in distinct wine-producing areas, morpho-anatomical and physiological differences among cultivars should also be further explored, as they can give new insights on the extent of varietal resilience or tolerance to abiotic stress conditions, to better understand cultivar responses to the changing climate in order to support viticulture adaptation in arid and semiarid regions for the near future (Ferrandino et al., 2023).

In this work, we conducted a 3-year experimental trial on potted vines of two red grapevines cultivated in Sardinia—Cannonau (syn. Grenache) and Carignano (syn. Carignan)—exposed to prolonged heat stress conditions, in the presence or in the absence of UV radiation, compared to a control directly exposed to the natural environment in order to evaluate the physiological, biochemical, anatomical, and ultrastructural leaf responses, the ability of the two cultivars to develop heat stress tolerance strategies, and the morpho-anatomical and physiological traits involved for each distinct genotype × environment combination. The phenotyping approach allowed the characterization of the extent of differences in cultivar responses to heat stress, either in the presence or in the absence of UV-B light, as well as to describe the adaptive divergence of two genetically distinct grapevine varieties to the long-term interaction of both abiotic factors.

## 2 Materials and methods

### 2.1 Experimental setup and design

**Figure 1:**
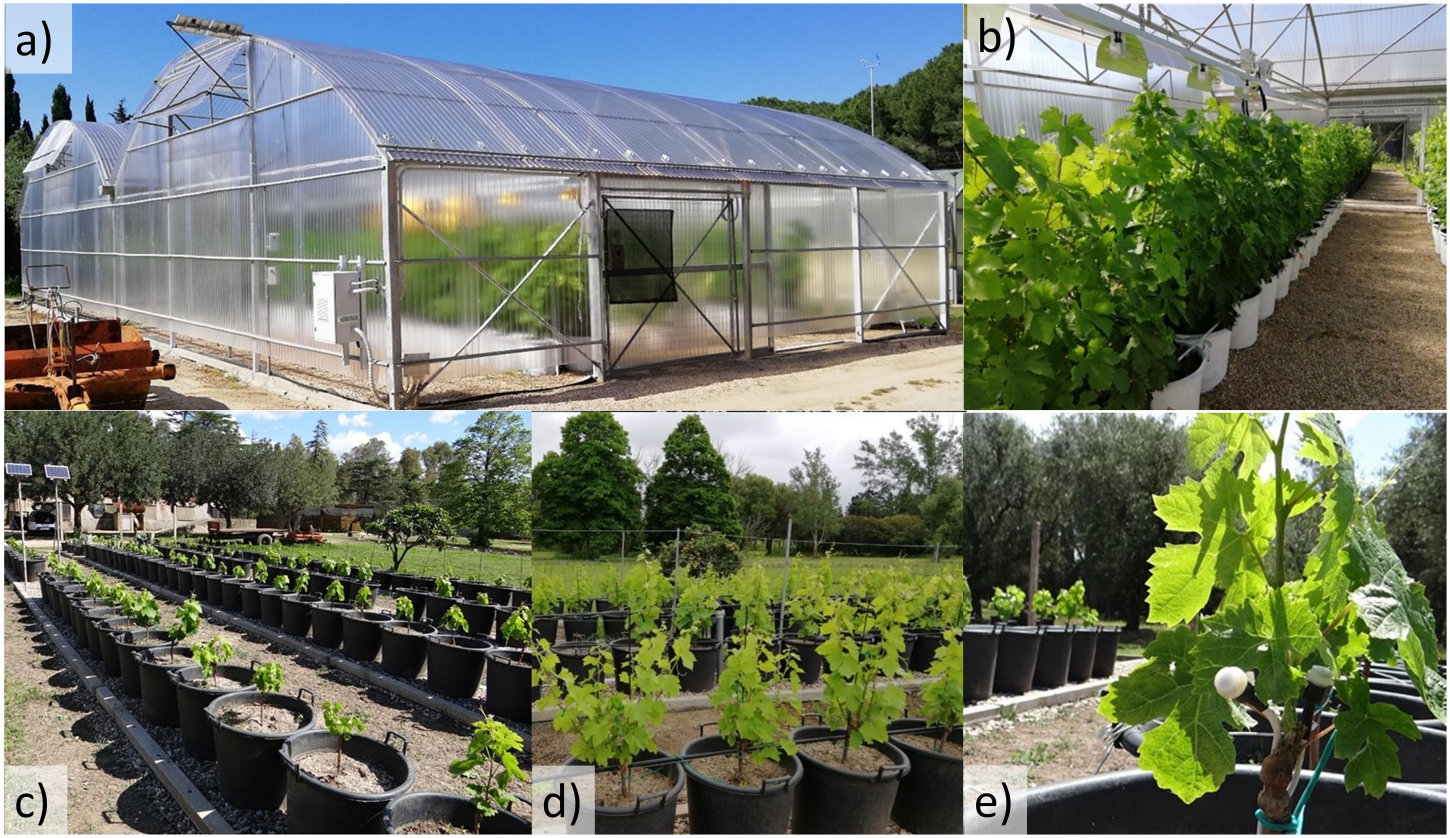
Illustration of the UV-screening greenhouse divided in two modules—+UV and −UV **(A, B)**—and the control plants in the experimental field **(C, D)**. Details of greenhouse plants distributed in rows **(B)**, in the UV and −UV treatments, and in the outdoor control **(C, D)** during the early phenological stages (BBCH 11–19) and of Rg and PAR radiation sensors at the base of a young shoot **(E)**.

The study was conducted during three growing seasons, from 2017 to 2019, on potted red grapevines (*Vitis vinifera* L.) cv Cannonau and Carignano, grafted on 1103 Paulsen rootstock in 2016, at the experimental farm of the University of Sassari in Oristano, Italy (39°54′11.87″ N, 8°37′7.62″ E at 13 m.a.s.l.). The climate of the study area is typically Mediterranean. The annual precipitation is approximately 540 mm; the minimum, maximum, and average daily solar exposure are approximately 6.8, 26.1, and 15.7 MJ m^−2^, respectively; and air temperatures (*T*_air_) range from average maximum values of 30°C during the hottest month and minimum averages of 7°C during the colder month (Fernandes de Oliveira et al., 2019). The experimental blocks were conducted on 2-year-old plants (40 plants per treatment and variety) potted on 50-L containers, with the soil substrate composed of fine soil, peat, and perlite (2:1:2). During the first year, all pots were colored white, and a single shoot was maintained and trained vertically along the trellis, supported by a vertical trellis with two parallel movable wires (**Figure 1**). In the 2nd and 3rd years, two and three vertical shoots were kept after spur pruning, and the canopy was topped frequently to keep the height of the vegetative wall within 2 m. In the spring of 2017, two blocks of plants of the two varieties were placed in a greenhouse (20-m length, 10-m width, 5-m maximum roof height, and 2.5 m at the gutter) with a metal support structure and covered with UV-blocking corrugated polycarbonate panels (Omega 76/15, 0.8 mm Suntuf Plus clear embossed, Palram Industries Ltd., Ramat-Yohanan, Israel), with 80% solar photosynthetically active radiation (PAR) transmission. Two doors located on the north and south sides, two side vents on the east side, and two circulation fans at 4 min height in the west promoted ventilation (**Figure 1**). The Cannonau and Carignano plants were arranged in adjacent rows, spaced 2.5 m apart, with north–south orientation. The greenhouse was divided in two modules of 80 plants each, separated by a UV-shielding polycarbonate sliding door. The northern module was equipped with a UV-B lighting system (Narrowband TL 100W/01 SLV/10, Phillips Lighting Holding B.V., Amsterdam, NL), suspended on a longitudinal track of adjustable height, above each plant row (treatment +UV). The daily UV-B dose was supplied throughout the growing seasons, between 9.00 h and 11.00 h, was 4.63 kJ m^−2^ d^−1^, while the environmental daily dose at the site, measured in June in the control block, was approximately 4.05 kJ m^−2^ d^−1^, and it was approximately 0 kJ m^−2^ d^−1^ in the south greenhouse UV-blocking module (−UV treatment). A PAR lighting system with high-pressure sodium lamps (Master GreenPower CG T 400W E40 1 SL/12, Phillips Lighting Holding B.V., Amsterdam, NL) ensured that, during the early morning hours (7.00 to 9.00 h), PAR photon flux density inside the greenhouse reached similarly high values simultaneously with the control plants (control, C) exposed to direct solar radiation (**Figure 1**). The PAR radiation flux was supplied both to the north and the south experimental block in the greenhouse. Irrigation was supplied with a surface, single-line drip irrigation system (4 l h^−1^), in order to keep high water availability from budburst to cluster closer (BBCH 9 to 80) and mild to moderate water stress thresholds (Ojeda, 2007) during the fruit developmental stages (BBCH 80–89). Irrigation was applied two to three times per day to avoid deficient drainage problems, with a 30-min maximum duration during the hottest periods (i.e., approximately 6 L plant^−1^ day^−1^).

**Figure 2:**
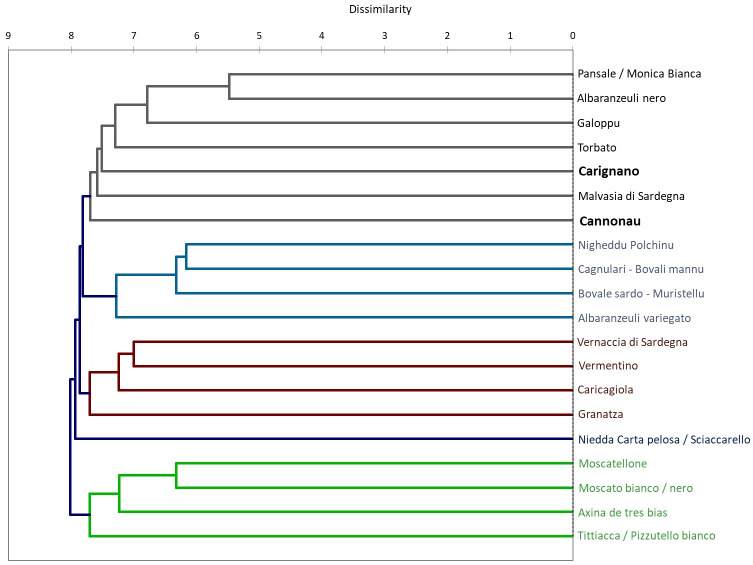
Dissimilarity dendrogram of the genetic distance among 20 traditional Sardinian varieties through agglomerative hierarchical clustering based on the comparison of 20 SSR profiles with 1,000 bootstrap samples.

The two varieties are among the most important red grape varieties grown in Sardinia: Cannonau is widespread throughout the territory, occupying as much as 29% of cultivated areas, while Carignano is typically grown in hot, arid, and highly sunny environments and prone to water and heat stresses only in 7% of the viticulture areas (Nieddu, 2011). They have very different ampelographic and physiological characteristics (Cipriani et al., 2010; Nieddu, 2011; Fernandes de Oliveira and Nieddu, 2015b). Cannonau plants have glabrous or slightly tomentose shoots, small and glabrous leaves, and shorter internodes, with a lighter green color compared to Carignano. The latter has highly tomentose, green shoots with the dorsal face of the internodes streaked with red color. The leaf is large and wedge-shaped; the upper leaf blade is slightly tomentose, while the lower is bristly (Nieddu, 2011). Compared to Cannonau, Carignano develops less secondary leaf area (Fernandes de Oliveira and Nieddu, 2016b; Fernandes de Oliveira et al., 2019). The two varieties showed different physiological behavior under water deficit: Cannonau exhibits tighter stomatal control over transpiration and higher constancy in minimum water potentials, while Carignano shows higher stomatal conductance and progressively lower minimum water potential. The varieties present quite different phenolic profiles (Vacca et al., 2009), and lower berry anthocyanin contents can be found in Cannonau, especially during a hot and dry ripening season (Fernandes de Oliveira and Nieddu, 2016b). An important genetic distance was shown in previous studies (Lovicu, 2017). Among the group of traditional Sardinian varieties, the genetic distances between Cannonau and Carignano was estimated by agglomerative hierarchical clustering based on a comparison among 22 SSR profiles, confirming the genetic diversity between the two varieties, which had only 36% of alleles in common (**Figure 2**) (Pelsy, 2010; Lovicu, 2017).

### 2.2 Meso and microclimatic conditions and water balance

**Figure 3:**
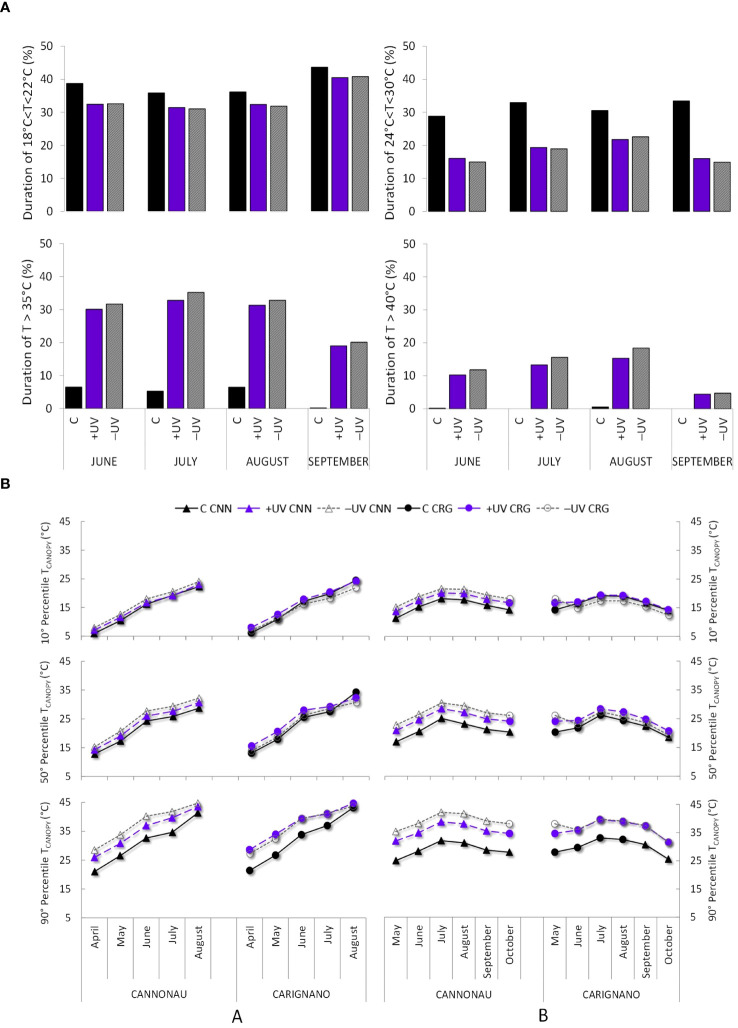
**(A)** Percent duration of low, mean, high, and very high air temperature (*T*_air_) ranges—18°C–22°C, 24°C–30°C, > 35°C, and > 40°C—from June to September 2019. **(B)** Canopy temperature (*T*_canopy_) average percentile 10°, 50°, and 90°from April to August 2018 and from May to October 2019 in the two varieties (CNN, Cannonau; CRG, Carignano) and in the three treatments (C, +UV, and −UV).

Meteorological data from the closest weather station (distance = 250 m) were used to assess the monthly rainfall, average daily solar radiation, and daily maximum, average, and minimum temperatures over the seasons. The weather conditions over the three seasons were compared with the climatic 30-year reference period (https://www.sar.sardegna.it/). **Supplementary Table S1** reports the monthly average temperatures and accumulated precipitation during each of the three seasons of trial and the monthly average of the reference historical series. The mean values of the study years were compared with the climate series (**Supplementary Table S1**). Canopy temperature (*T*_canopy_), incoming global solar radiation (Rg), and PAR were continuously recorded on three plants of each treatment and variety, from the beginning of the growing season until harvest, at 10-minute intervals using temperature microprobes (MTP microprobes, GMR instruments, FI, IT) and radiation sensors coupled to dataloggers (GMR instruments, FI, IT), placed on an internode at mid-shoot height, close to a lower leaf blade, and fixed with polytetrafluoroethylene tape. The Rg and PAR sensors were calibrated using a pyranometer and a PAR sensor (Skye instruments, Ltd., Llandrindod Wells, UK) to check the accuracy on solar irradiance assessment under field conditions. The PAR and Rg sensor calibration curves and hourly average values of Rg, PAR, UV-A, and UV-B measured on a sunny day of June, outside and inside the greenhouse, using individual sensors connected to a portable SpectroSense+ datalogger (Skye Instruments, Llandrindod, Wells, UK) are presented in **Supplementary Figures S1**, **S2**, respectively. All sensors were placed at the middle part of the shoots in early growth stages (**Figure 1**) and thereafter kept within 60 cm of the apex. **Supplementary Figure S3A, B** reports the daily UV-B supplied in the C and +UV module of the greenhouse, measured in the plants, taking into account the sensor calibration curves and the vertical fields of irradiance attenuation under UV lamp (Aphalo et al., 2012). Sunlight intensity inside and outside the greenhouse was also measured with a portable ASD spectroradiometer (FieldSpec 4 Hi-Res 350–2,500 nm, Malvern Panalytical, Westborogh, MA, USA) (**Supplementary Figure S3C**). The duration of low (18°C–22°C), mean (24°C–30°C), high (>35°C), and very high (>40°C) *T*_air_ ranges in C, +UV, and –UV environments, respectively, was computed (**Figure 3A**) based on the data recorded by an ATMOS 14 T/RH, monitoring system inside a radiation shield positioned at 2 m in height between vine rows and connected to a Em50 datalogger (Meter Group Inc., Pullman, WA, USA). The 10, 50, and 90 percentiles of exposure to different *T*_canopy_ ranges throughout the study periods were calculated to summarize the differences in thermal environment among the three treatments throughout the growing season (**Figure 3B**). Canopy temperature depression (*T*_air_ – *T*_canopy_) was evaluated for each variety × treatment combination, expressed as average difference between *T*_air_ and *T*_canopy_ along the day (**Supplementary Figure S4**). Phenological succession was recorded weekly in each treatment, and the accumulated thermal time for each phenophase was compared using the Normal Heat Hour model (Mariani et al., 2012).

### 2.3 Water status, individual leaf gas exchange, and chlorophyll fluorescence

Stem water potential was monitored in the morning (8.30 h) and at solar noon (13.00 h) on mid-canopy adult leaves during veraison (BBCH 81) using a portable pressure chamber (Pump up, PMS Instruments, USA). Prior to the measurement, the leaves were enclosed for 1 h in plastic-coated aluminum bags to allow balancing leaf water status with that of the stem. On the same day, daily leaf gas exchange was monitored at 8.30, 10.30, 13.00, and 16.00 h using an infrared gas analyzer (CIRAS−3) coupled with a Parkinson’s leaf chamber for single leaf measurements. Net assimilation rate, stomatal conductance, transpiration rate, intrinsic water use efficiency leaf temperature (*T*_leaf_), leaf vapor pressure (*e*_a_), and the vapor pressure deficit (VPD) between the leaf and the air were measured on fully expanded, well-exposed leaves, keeping the reference air within a CO_2_ concentration of 400 µmol mol^−1^ and 50% relative humidity. A standard photosynthetic photon flux density of 1,000 µmol m^−2^ s^−1^ was imposed during the measurements by connecting a LED light unit to the leaf chamber.

Direct chlorophyll fluorescence of PSII was monitored *in vivo* on fully expanded leaves from pea size (BBCH 75) until the beginning of senescence and leaf color change (BBCH 92). After a dark adaptation period of 30 min, fluorescence transients were recorded using a Handy PEA fluorimeter (Hansatech Instruments Ltd., King’s Lynn, UK), after applying an actinic saturating red light (650 nm) intensity of 3,000 µmol m^−2^ s^−1^, in single flashes, with the signal gain at 1.0 and a 30-s duration in each replicate. Rapid fluorescence kinetics from the minimum (*F*_0_) to the maximum (*F*_m_) fluorescence of dark-adapted leaves was recorded at a 10-µs time step, allowing for main and derived fluorescence variables calculation (**Table 1**) and thus for evaluating PSII performance during the photochemical and thermal phases of the fluorescence transient (Strasser et al., 2000). The main derived variables analyzed were as follows: maximum yield of primary photochemistry of PSII, φP_o_; maximum water splitting efficiency, WSE; quantum yield for electro transport, φ_eo_; quantum yield for energy dissipation, φDI_o_; density of the reaction centers, RC_QA_; and the linear indicator of functional PSII units, 1/*F*_0_ − 1/*F*_m_ (Flexas et al., 2001; Strasser et al., 2007). After the direct measurements on intact leaves, PSII chlorophyll fluorescence was also monitored on leaf samples collected for lab analysis.

**Table 1:** Mean values (*n* = 6) and two-way ANOVA significance of the effects of cultivar, treatment, and their interaction on midday leaf photochemical performance from fruit set until beginning of senescence (BBCH 73–92) in season 2018.

DOY/BBCH	CV	Treatment	QP_0_	QDI_0_	1/*F*_0_ – 1/*F*_m_	Qe_0_	WSE	RC_QA_
163/73	CRG	C	0.79	0.21	0.0018 b	0.29	3.82	590.3 b
		−UV	0.74	0.26	0.0029 a	0.31	2.83	314.1 c
		+UV	0.77	0.23	0.0016 b	0.33	3.63	650.9 a
	CNN	C	0.79	0.21	0.0018 b	0.30	3.80	598.9 b
		−UV	0.78	0.22	0.0016 b	0.30	3.62	645.2 a
		+UV	0.68	0.32	0.0021 ab	0.37	2.54	460.6 c
	Sign.	CV	0.574	0.575	0.251	0.735	0.799	0.228
		Treatment	0.225	0.225	0.302	0.490	0.339	0.085
		Interaction	0.202	0.203	0.028	0.907	0.178	0.0001
184/80	CRG	C	0.77	0.23	0.0016	0.33	3.46	587.0 a
		−UV	0.72	0.28	0.0014	0.29	2.65	515.3 b
		+UV	0.74	0.26	0.0016	0.31	3.05	562.1 b
	CNN	C	0.80	0.20	0.0017	0.46	4.08	813.6 a
		−UV	0.76	0.24	0.0016	0.31	3.24	505.9 b
		+UV	0.69	0.31	0.0013	0.29	2.39	435.0 b
	Sign.	CV	0.970	0.970	0.810	0.443	0.369	0.762
		Treatment	0.097	0.097	0.391	0.223	0.526	0.015
		Interaction	0.317	0.317	0.317	0.470	0.461	0.051
214/83	CRG	C	0.83 a	0.17 c	0.0018 a	0.40 a	4.74 a	797.3 a
		−UV	0.78 b	0.22 b	0.0017 a	0.41 a	3.80 b	681.8 b
		+UV	0.73 b	0.27 b	0.0014 b	0.26 b	2.78 c	496.7 c
	CNN	C	0.59 c	0.41 a	0.0009 c	0.25 b	1.81 c	438.9 c
		−UV	0.77 b	0.23 b	0.0017 b	0.31 ab	3.39 b	557.6 b
		+UV	0.83 a	0.17 c	0.0020 a	0.38 ab	4.93 a	735.7 a
	Sign.	CV	0.215	0.215	0.416	0.139	0.390	0.315
		Treatment	0.230	0.230	0.086	0.545	0.584	0.999
		Interaction	0.007	0.007	0.004	0.004	0.002	0.026
249/89	CRG	C	0.82 a	0.18 b	0.0019 a	0.39	4.67 a	741.2 a
		−UV	0.69 b	0.31 a	0.0013 c	0.28	2.28 c	422.4 c
		+UV	0.77 ab	0.23 ab	0.0015 b	0.30	3.40 b	672.0 b
	CNN	C	0.78 ab	0.22 ab	0.0016 a	0.30	3.49 b	572.6 b
		−UV	0.75 b	0.25 a	0.0015 b	0.29	3.08 c	543.3 b
		+UV	0.80 a	0.20 b	0.0016 a	0.39	4.04 a	764.3 a
	Sign.	CV	0.395	0.395	0.861	0.888	0.735	0.808
		Treatment	0.001	0.001	0.013	0.260	0.002	0.021
		Interaction	0.021	0.021	0.007	0.104	0.013	0.126
284/92	CRG	C	0.73	0.27	0.0015	0.26	2.99	443.0
		−UV	0.79	0.21	0.0018	0.36	3.71	537.6
		+UV	0.77	0.23	0.0016	0.40	3.36	646.5
	CNN	C	0.76	0.24	0.0019	0.37	3.22	437.4
		−UV	0.63	0.37	0.0018	0.27	2.66	396.2
		+UV	0.78	0.22	0.0017	0.36	3.78	706.7
	Sign.	CV	0.542	0.542	0.371	0.895	0.809	0.746
		Treatment	0.637	0.637	0.660	0.560	0.743	0.093
		Interaction	0.386	0.386	0.527	0.342	0.481	0.642

Different lowercase letters indicate significant differences among treatments.

### 2.4 Leaf optics and pigments

Based on chlorophyll fluorescence screening method (Goulas et al., 2004), leaf chlorophyll, CHL, anthocyanin, ANT, flavonol, FLA e nitrogen balance index, and NBI (Cartelat et al., 2005; Diago et al., 2016) were estimated *in vivo*, in field, and in laboratory adult leaf samples from the middle shoot height using Dualex scientific+ (Force-A, Paris, FR), a portable leaf clip analyzer that measures the transmittance and screening effect of polyphenol chlorophyll fluorescence (Cerovic et al., 2012). The measurements were performed during flowering (BBCH 65), pea size (BBCH 73), cluster closure (BBCH 80), veraison (BBCH 83), and in 6 days of the year (DOY), from May to October 2018, on six adult leaves per treatment and variety with five reads in each leaf blade side. The measurements were repeated in the laboratory, and the leaf area of each replicate was estimated (Fernandes de Oliveira and Nieddu, 2016a; Fernandes de Oliveira et al., 2021). Furthermore, leaf mass per area (LMA) was estimated on 5-cm-diameter leaf blade discs, dried at 60°C for 48 h, and weighed. Then, leaf relative water content (RWC) was calculated. All the leaf samples used for spectrophotometric measurements were previously washed and dried at room temperature.

Spectral reflectance was measured using a FieldSpec 4 Wide-Res spectroradiometer (range, 350–2,500 nm; ASD Inc., Boulder, CO, USA) coupled to a plant probe with leaf clip for live vegetation. Each spectral measurement was the average of 10 spectra, keeping the black standard under the leaf sample (*n* = 6). The measurements were preceded by a dark current measurement and a white reference measurement. Five independent measurements were taken on each sample on both adaxial and abaxial leaf blades, and the average reflectance of each leaf was then calculated. Thereafter, the reflectance spectra were normalized at the reciprocal reflection spectrum of chlorophyll a (678 nm) to highlight the different proportion of pigments as compared to chlorophyll a (Merzlyak et al., 2003; 2008). Other reflectance indexes were calculated in order to characterize specific differences among treatments and varieties related to leaf composition and structural traits, namely—the normalized difference chlorophyll index, NDCI, for chlorophyll estimation (Gitelson and Merzlyak, 1994; Mishra and Mishra, 2012); TCARI/OSAVI (Daughtry et al., 2000; Wu et al., 2008); the modified anthocyanin reflectance index, mARI (Gitelson et al., 2009); the normalized difference index for leaf area mass per area, ND_LMA_; and the normalized dry matter index, NDMI (Cheng et al., 2014).

Leaf colorimetric estimation of chlorophyll, anthocyanin, and total polyphenol contents was carried out using a CARY 50 Scan UV-Vis Varian spectrophotometer (Agilent Technologies, Santa Clara, CA, USA), following the method of Lichtenthaler (1987), after the extraction of compounds from 15 discs (of 16-mm diameter) on 10 mL methanol solution. Finally, chlorophyll a (Chl a), chlorophyll b (Chl b), and carotenoid (Car) contents were derived as proposed by Lichtenthaler and Wellburn (1983). Total phenols were determined with Folin–Ciocalteu colorimetric method using a solution of 100 µL extract. The absorbance readings were taken at 750 nm using Hitachi model 100**–**60 spectrophotometer (Hitachi High-Technologies Corporation, Tokyo, Japan). Total phenol content was expressed in terms of catechin concentration, mg m^-2^. For anthocyanin determination, the Di Stefano and Cravero (1991) methodology was applied. In addition to the fresh weight of each leaf sample, dry weight was determined after drying in an oven at 105°C for more than 24 h until a constant mass value was reached. Thereafter, the samples were pulverized for carbon, hydrogen, and nitrogen content determination using an infrared EN CHN 628 series elemental analyzer (LECO Corporation, St. Joseph, MI, USA).

### 2.5 Leaf morpho-anatomy

In each treatment and variety, six adult leaves were collected from the outer leaf wall of the canopy, sealed in plastic bags, and transported to the laboratory for morphological and anatomical analyses. The leaves were washed, cleaned, scanned, and processed with Image J software (https://imagej.net/) to measure the main leaf vein length. Leaf swelling spots were delimited in each leaf image, using freehand selections, to calculate the percentual leaf swelling area in each leaf area. Then, leaf cross-sections were collected, aside from the main vein, with a razor blade. The sections were colored using two differential stains applied successively, iodine green and carmine alum, mounted on glass slides, then washed and covered with adhesive tape, and dehydrated with increasing concentrations of ethyl alcohol solutions (50%, 75%, 90%, and 100%). The images of the leaf cross-section were captured with an Infinity digital camera (Lumenera Corporation, Ottawa, ON, CA) mounted on a Zeiss Axiophot optical microscope (Zeiss Microscopy, Oberkochen, DE). Leaf thickness, mesophyll thickness, and percentage of mesophyll cross sectional area occupied by palisade and spongy mesophyll were observed and measured with the ×20 objective. Stomata and intercellular chamber were also observed in leaf cross-section images.

In order to observe further morphological differences in leaf tissues, namely, organelle structure, metabolite deposition, and overall cellular activity, leaf blade cross-sections (3 × 3 mm) were excised from leaves in each variety and treatment, fixed with 2.5% glutaraldehyde in 0.2 M sodium phosphate buffer (pH 7.2), and post-fixed with 1% osmium tetroxide in the same buffer for 1 h. The samples were then dehydrated in graded acetone series and embedded in Agar100 epoxy resin; ultra-thin sections were cut on an ultramicrotome RMC MT-7 using a diamond knife and stained with uranyl acetate and lead citrate. The sections were then examined in a Zeiss EM109 transmission electron microscope operating at 80 kV (Zeiss Microscopy, Oberkochen, DE).

### 2.6 Statistical analysis

Two-way analysis of variance (ANOVA) and least significant difference (LSD) test were performed on all data, using the SPSS 25.0 software (SPSS Inc., Chicago, IL, USA), in order to compare means and detect significant differences among treatments and cultivar at the 95% confidence level as well as to highlight the statistically different interaction effect among the two main factors leading to plant susceptibility and adaptive responses to light and heat stresses.

## 3 Results

### 3.1 Light, thermal, and thermo-hygrometric environment

The most relevant weather records during the 3 years of study concerned the systematic increases in maximum temperature during the summer months, from 2017 to 2019 (the anomalies ranging from +3.2°C to +5°C), and in 2018 the maximum temperatures were above the climatic 30-year average from April until July (+3.2°C and 2.8°C). As far as the light microclimate is concerned, the solar irradiance transmitted inside the greenhouse, measured in June during a clear day, was significantly different in both UV-A, visible, and infrared wavelengths (350 to 1,000 nm) compared to the outdoor environment (**Supplementary Figure S3C**). For what concerns the visible wavelengths, the solar irradiance transmitted inside the greenhouse at midday was similar to that recorded outside by midmorning and approximately 11% lower than that measured outside in the early afternoon and 12% higher than that measured in the greenhouse at midmorning. The average irradiance in near-infrared wavelengths (760–1,000 nm) was doubled inside the greenhouse compared to that which occur naturally (about double in 960–1,000-nm wavelength; 0.31 vs. 0.15 kW m^−2^, respectively). The Rg intercepted by the canopies inside the greenhouse was reduced by ca. 24% due to the foliage filtering effect (**Supplementary Figure S3A**). On average, UV-B radiation in the inner canopy (at ca. 60 cm) reached similar percent values in +UV plants as those intercepted by the control canopies exposed to direct solar radiation. Finally, the daily average canopy Rg and UV-B intercepted and the daily average accumulated Rg and UV-B in control and greenhouse plants are shown in **Supplementary Figure S3B**. Inside the greenhouse, the +UV and −UV canopies intercepted ca. 1,539 W m^−2^ of Rg per day, i.e., approximately 76% of the Rg measured in control canopies during the summer.

The thermal environment along growth stages was characterized by a percent duration of low *T*_air_ (18°C–22°C) inside the greenhouse of 33%−35% during June, July, and August and of 40% in September, while in the control environment such temperatures were recorded for a 35% duration in July and August and approximately 40%−45% in June and September (**Figure 3A**). In the greenhouse, *T*_air_ ranges of 24°C to 30°C did not exceed 18−20% duration, while in the outdoor control such temperatures were recorded for as much as 30%−35% season duration. The most important differences between environments regarded the permanence of high (>35°C) and very high *T*_air_ (>40°C). Overall, the thermal trends in both greenhouse modules, +UV and –UV, were similar throughout the experimental trial. As far as plant thermal microclimate is concerned, **Figure 3B** shows the *T*_canopy_ trends from May to August 2018 and from April to October 2019, expressed in terms of 90th, 50th, and 10th percentiles. The *T*_canopy_ of C plants, in both varieties, was always lower than that of the plants under +UV and –UV treatments, except for the beginning of August 2018 when a prolonged extreme heat wave was recorded, resulting in a sharp increase in heat stress conditions even in the control potted plants. Throughout the duration of the trial, the Cannonau plants subjected to the –UV treatment reached higher *T*_canopy_ than the Carignano plants. In 2019, the differences in *T*_canopy_ among control and greenhouse treatments became more pronounced; the plants in the greenhouse recorded significantly higher *T*_canopy_ than that under control conditions in both varieties. Though there was no strong upward trend in temperature along the season, the *T*_canopy_ inside the greenhouse was 10°C higher than those outside for 90% of the temperature regime and 3°C−5°C higher in the greenhouse than in the control for 50% of the recorded *T*_canopy_ and 1°C−2°C higher in the greenhouse than in the control in 10% of these records. Compared to the previous season, the maximum differences in *T*_canopy_ between the control and the two treatments in the greenhouse nearly doubled in 2019. The differences between +UV and –UV treatments inside were also not evident in Carignano, while they were quite different in Cannonau for which the –UV plants had warmer canopies than that under +UV treatment. Regarding average daily thermohygrometric conditions, the greenhouse and control environments were markedly different. On average, the maximum *T*_air_ values recorded between 12.00 and 16.00 h were approximately 30°C, and air RH was 50%, while in the greenhouse average *T*_air_ was 40°C with a RH of 40%. The overnight *T*_air_ values were similar in all treatments, with the lower values reaching 20°C and with approximately 80% of RH (**Supplementary Figure S4**). Thus, +UV and –UV treatments were characterized by the persistence of high *T*_air_, large day–night ranges of *T*_air_ (**±** 20°C), and RH (**±** 40%).

### 3.2 Phenology and accumulated thermal time

**Figure 4:**
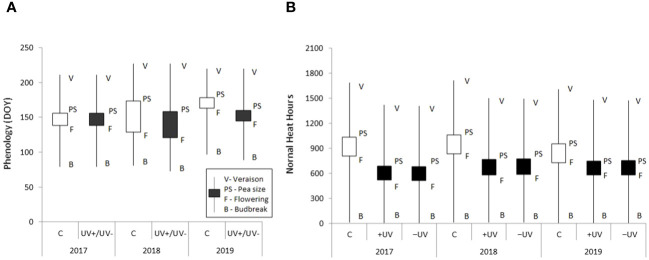
**(A)** Boxplots showing the main average (*n* = 6) phenological stages of canopy development during the study, monitored on control and greenhouse plants (+UV/−UV). **(B)** Boxplots of the accumulated thermal time for the phenological courses using the Normal Heat Hours model based on air temperature data in the greenhouse and in the control plot.

The duration of each phenological stage was not significantly different among the three environments (**Figure 4A**). Nevertheless, the year effect on the duration of phenological stages was quite pronounced, particularly on the duration of flowering and berry growth stages. In 2017, no phenological advances from bud break until veraison were observed inside the greenhouse as treatment differentiation began shortly before bud break, yet the low chilling requirements of grapevines to overcome endormancy, together with the increased accumulated heat units for vegetative growth restart inside the greenhouse in 2018, resulted in a cumulative advance of 8 days in bud break and successive growth stages as compared to control plants (**Figure 4A**). The influence of interannual meteorological differences on the phenological stage occurrence and duration was higher than that of the meso and microclimatic differences among treatments. These included a delay of 16 days in bud break 2019 and a 24-day delay in flowering compared to 2018 (**Figure 4B**). Moreover, flowering duration was much shorter, nearly half, in all treatments in 2019. In fact, flowering duration was the most sensitive stage to inter-annual weather variations, and higher uniformity of successive fruit growth interphases was observed (only 5- and 2-day difference in pea size date in control and greenhouse, respectively). A marked effect of *T*_air_ in the occurrence of veraison was evidenced, with a 7-day earlier onset in 2019 compared to 2018. The Normal Heat Hours model enabled an accurate classification of the local thermal resources for grapevine development and illustrated the prolonged warming effect of the greenhouse on phenological successions, particularly during summer.

### 3.3 Vegetative growth, physiological performance, and leaf composition

As expected, plant growth was stimulated in the protected environment, with intense shoot and leaf area development (**Supplementary Figure S5A**) that was kept at a height lower than 2.5 m with frequent topping operations. For this reason, up to the third year of the experimental trial, the vigor of control shoots at pruning was not statistically different from that of the greenhouse plants in Cannonau, yet the control plants of Carignan showed significantly lower vigor than greenhouse plants (**Supplementary Figure S5B**). Similarly, an increasing trend in average main leaf area development in Carignano plants subjected to C, –UV, and +UV was recorded, with an average value of 104, 207, and 249 cm^2^ in Carignano compared to 48, 59, and 58 cm^2^ in Cannonau, respectively. The average main leaf size was significantly larger (approximately double) in the –UV and +UV treatments (133 and 154 cm^2^, respectively) than in C (approximately 75 cm^2^). From cluster closure (BBCH 80) onward, the differences between the two greenhouse treatments became significant, with the +UV plants showing twice as much single leaf area as the –UV and approximately four times the leaf area of control leaves. Conversely to Carignano, the differences in average main leaf size were not statistically significant in Cannonau, and this variety showed less development of single main leaf area from flowering to harvest (BBCH 74–89).

**Figure 5:**
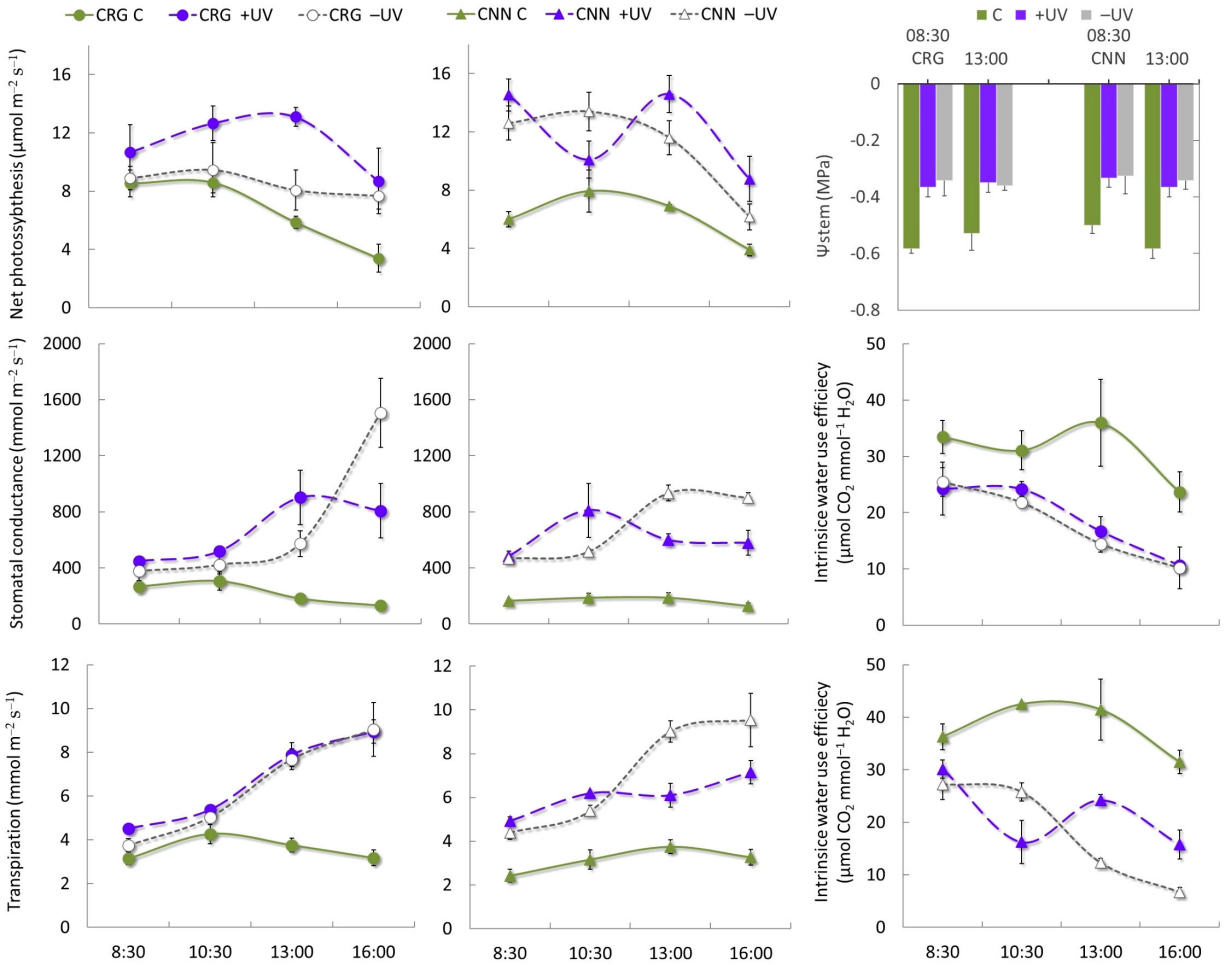
Daily pattern of leaf gas exchange and early morning and midday stem water potential at veraison (BBCH 81) 2018 in Cannonau (CNN) and Carignano (CRG) plants subjected to C, +UV, and −UV treatments. Mean values (*n* = 6) ± standard error.

From bud break to the onset of ripening (BBCH 9–80), the plants were kept under high soil water availability, and stem water potential did not fall below –0.6 MPa. Irrigation supplies kept plants under mild water stress, thus not limiting photosynthetic performance and efficiency (**Figure 5**), even during the afternoon, although *T*_leaf_ exceeded 35°C and *T*_air_ remained very high for a long time (**Figure 3A**). The pattern of net assimilation rate over a hot day of veraison evidenced differences among varieties in response to the different *T*_air_ and light microclimate among treatments. During the morning, net photosynthesis reached higher values inside the greenhouse, and significantly lower values were recorded in Cannonau control plants in early morning but not in Carignano, and by midmorning the differences among treatments and varieties were significant. These results can be ascribed to the PAR supplied in early morning and the higher photosynthetic performance of leaves under high diffuse PAR environments. By midday and in the afternoon, net photosynthesis decreased, and the differences between treatments and varieties were further attenuated. During the morning, Carignano plants showed similar stomatal conductance and transpiration rates among treatments, while Cannonau exhibited a higher morning stomatal control over transpiration only in C plants. In C plants, stomatal conductance varied little over the day in the two varieties, with only a slight increase during the morning of Carignano (ca. 300 mmol m^−2^ s^−1^) as compared to Cannonau (less than 200 mmol m^−2^ s^−1^). Inside the greenhouse, stomatal conductance increased significantly but varied among similar values in both varieties, from approximately 400 mmol m^−2^ s^−1^ in the morning to an afternoon maximum that exceeded 1,600 and 1,200 mmol m^−2^ s^−1^ in –UV plants Carignano and Cannonau, respectively (**Figure 5**). These resulted in a sharp reduction of intrinsic water use efficiency, compared to C plants, that reached more than twice the values of greenhouse plants. However, Carignano +UV leaves were able to sustain significantly higher water use efficiency in the afternoon compared to the other plants inside the greenhouse. The transpiration rate over the day followed an increasing trend inside the greenhouse, with higher average values in the afternoon, though significantly lower in Cannonau +UV leaves compared to –UV.

The effects of the treatments on the primary photochemistry performances of dark-adapted leaf samples are presented in **Table 1**. At fruit set (BBCH 73), main differences regarded 1/F_0_ − 1/*F*_m_ and RC_QA_, the latter being higher in Carignano leaves subjected to +UV and in Cannonau –UV leaves. As the season proceeded, with increased high *T*_air_ and continuous exposure to the light treatments, the differences became more pronounced. Carignano C and Cannonau +UV plants exhibited higher QP_0_, with less investment in energy dissipation, and also higher PSII functionality, RC_QA_, Qe_0_, and WSE. Greenhouse treatments, particularly −UV, resulted in a significant reduction in the functional units of PSII, RC_QA_, and WSE measured at mid-ripening (BBCH 89). However, in Cannonau, the +UV treatment resulted in increased 1/F_0_ − 1/*F*_m_, WSE, and RC_QA_ compared to C. After ripening, the differences concerning leaf photochemical efficiency flattened, and as senescence began (BBCH 92) these differences disappeared.

**Figure 6:**
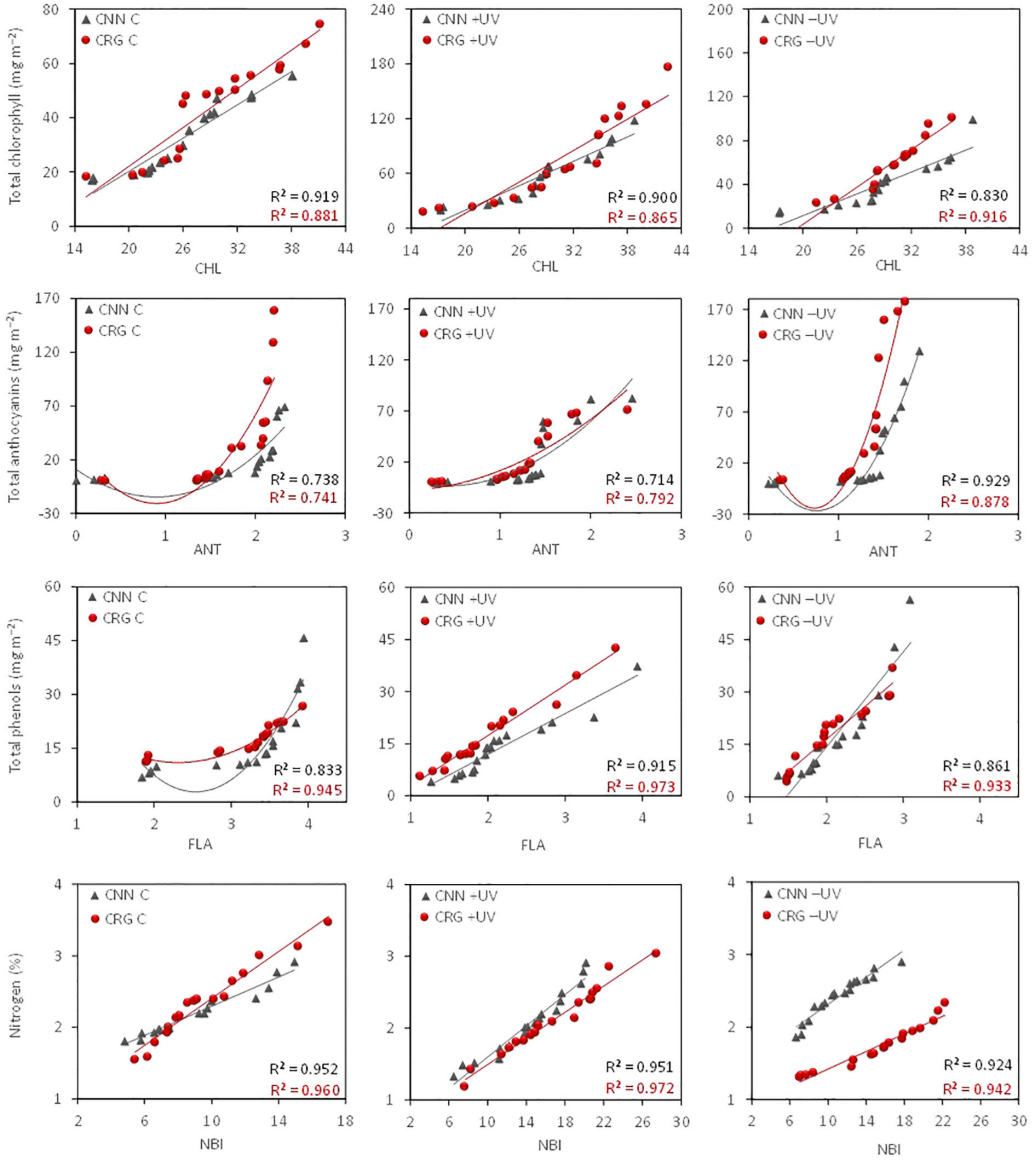
Correlation patterns and coefficients of the mean chlorophyll (CHL), anthocyanin (ANT), flavonol (FLA), and nitrogen balance (NBI) indices and mean chlorophyll, total anthocyanin, polyphenol, and nitrogen contents in the leaves of Cannonau and Carignano subjected to C, +UV, and −UV measured from fruit set until leaf senescence (BBCH 73–92) in five dates of growing season 2018.

The comparison of CHL, ANT, FLA, and NBI estimates with the relative leaf pigment and nitrogen content indicates a high correlation between estimated data and leaf compound quantification in the lab (**Figure 6**; **Supplementary Tables S2–S4**). The correlation patterns were different between varieties. However, the most pronounced differences were observed among treatments. As far as the nitrogen balance is concerned, data indicate a higher concentration on greenhouse leaves, with higher nitrogen contents per unit of leaf dry weight in +UV and −UV than in C leaves. Moreover, the average chlorophyll contents were significantly higher in the greenhouse than in the control plants and in Carignan rather than Cannonau, although the average values of CHl b/Chl a ratio for the whole season were not statistically different among treatments and varieties (**Supplementary Table S3**). Among the leaf compositional differences, water content and fresh weight were also significantly higher in Cannonau compared to Carignano and particularly in C compared to −UV and +UV. The −UV plants had lower turgid leaf weight compared to C and +UV in both varieties. Leaf dry matter was significantly higher in Carignano, and the leaf mass per single leaf was significantly higher in Carignano than in Cannonau, being also significantly lower in C and higher in +UV plants. The treatment effect was more pronounced in Carignano.

**Figure 7:**
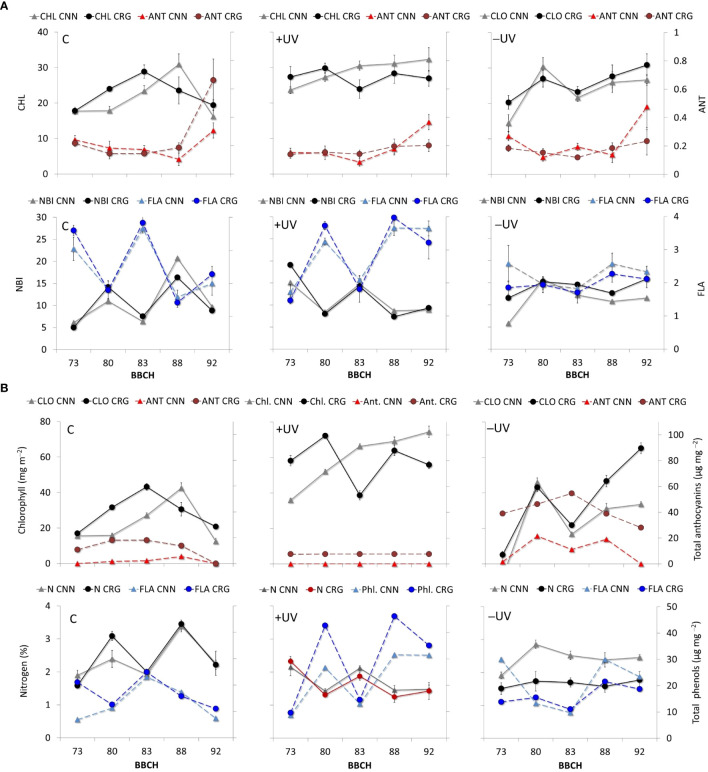
**(A)** Patterns of CHL, ANT, FLA, and NBI measured in Cannonau (CNN) and Carignano (CRG) leaves subjected to C, +UV, and −UV measured from fruit set [until leaf senescence (BBCH 73–92] in five dates of growing season 2018. **(B)** Patterns of total chlorophyll, anthocyanin, polyphenol, and nitrogen contents in leaves estimated based on the patterns leaf CHL, ANT, FLA, and NBI and the correlation patterns between the indexes and pigment contents in each variety and treatment.

The patterns of CHL, ANT, FLA, and NBI highlighted the effect of greenhouse in increasing and keeping high CHL values throughout the season in both varieties (**Figure 7A**). This effect was maximized in +UV plants at fruit set (BBCH 73). Along the season, fluctuations in CHL with *T*_leaf_ were observed in all inverse treatments and varieties, except for the measurements taken by leaf senescence (BBCH 92). The ANT measured on grapevine leaves varied among very low values over the season. Higher values were recorded by fruit set (BBCH 73) and during leaf senescence (BBCH 92) in both C and +UV plants. During three central dates, this index varied among similarly low values in every treatment and variety. In addition, FLA and NBI suggest significantly higher flavonol accumulation +UV leaf tissues, in both varieties though with large fluctuation both in FLA and NBI over the season. **Figure 7B** shows the dynamics of total chlorophyll, anthocyanin, phenols, and leaf nitrogen contents corresponding to the CHL, ANT, FLA, and NBI values shown in **Figure 7A**, as estimated based on the correlation patterns in **Figure 6**. When comparing the data measured in the field with that of leaf samples in the lab, the differences observed with optical estimation were amplified (**Figure 7B**). The chlorophyll and polyphenol estimates were markedly higher in +UV and smaller in −UV, and also the amplitude of variations along the season was higher. Anthocyanin estimates remained stably low in C and +UV, in Cannonau, while an important and consistent increase in estimated anthocyanins was observed in Carignano, in +UV, and even more markedly in −UV. In addition to the treatments, a different varietal response to the presence/absence of UV-B light was observed, characterized by increasing increment in chlorophyll estimates in Cannonau +UV along the season, even during the central period, for which the daily duration of high *T*_air_ was maximum. In contrast, Carignano +UV leaves showed a decrease in estimated chlorophyll during the same period.

**Figure 8:**
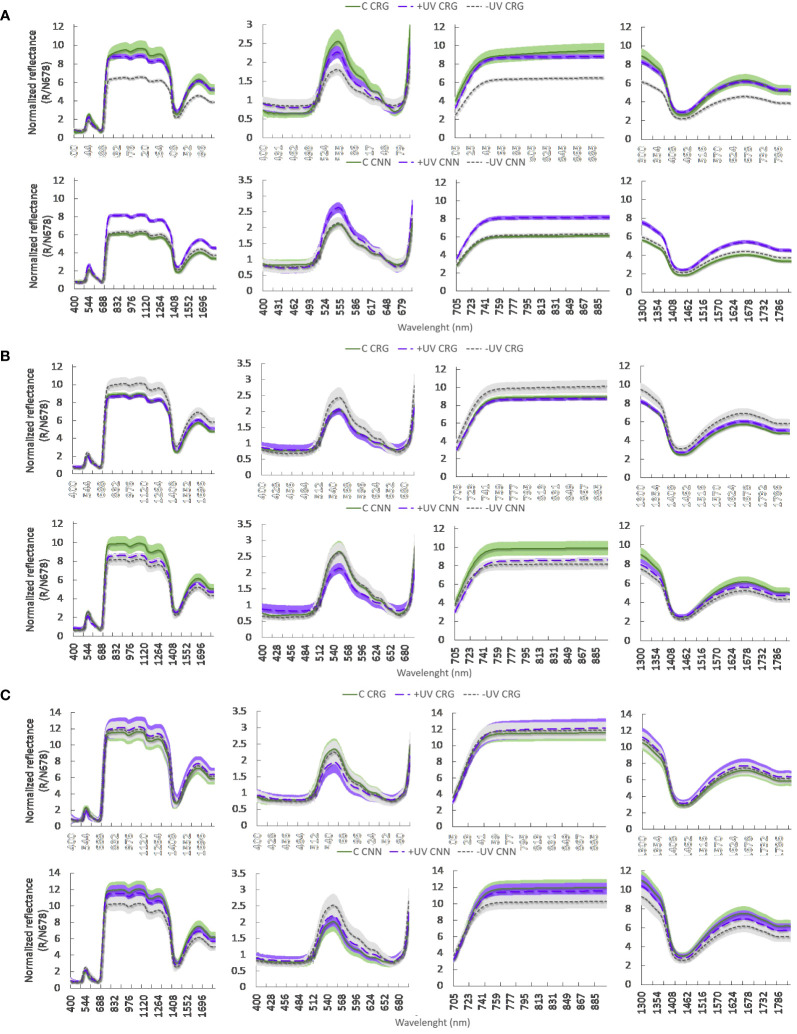
Leaf reflectance spectra normalized at the reciprocal reflection spectrum of chlorophyll a (R/N678) recorded in each variety (CNN, Cannonau; CRG, Carignano) and treatments in 2018 **(A)** during flowering, **(B)** at pea size, and **(C)** at early veraison (DOY 148, 164, and 184, respectively). The light color bands—green, purple and gray—represent the standard error for each treatment: C, +UV, and −UV, respectively. In each four panels per row the reflectance data concern, respectively, the whole spectrum (350–1,900 nm), the visible region (400–700 nm), the near-infrared wavelengths (705–900 nm), and the short-infrared wavelengths (1,300–1,800).

Different reflectance spectra, normalized at the reciprocal reflectance spectrum of chlorophyll a, were observed between treatments in the two varieties, and a different evolution of these spectra was recorded during flowering (BBCH 65), pea size (BBCH 75) and cluster closure (BBCH 80) (**Figure 8**). During flowering, Carignan plants in the greenhouse exhibited higher values of spectral reflectance in most of the radiant spectrum, including the green (530−590 nm) and especially the red (705−745 nm) regions of the visible wavelengths, the near-infrared (760−900 nm), and short-wave infrared, SWIR (1,570−1,650 nm) regions. In Cannonau, C and −UV plants exhibited much lower reflectance values, compared to Carignano, while the +UV treatment resulted in higher reflectance values similar to those recorded in Carignano +UV. At pea size stage, the spectral reflectance in the red and infrared was much higher in all treatments, being significantly higher in Carignano −UV and Cannonau C. At this stage, the significance of differences between treatments in the SWIR region was reduced in both varieties, and Cannonau C and −UV leaves had similar reflectance values in the green region, which were higher than in +UV. At veraison, data variability increased markedly, and differences between treatments in leaf reflectance lost statistical significance over the entire radiant spectrum. However, Cannonau leaves kept the trend of higher reflectance in the green region and lower reflectance in the red and infrared. Similarly, analyzing the values of the vegetation indices (**Supplementary Table S5**), it can be noted that, on the first measurement date, the varietal differences in the estimated contents of photosynthetic pigments with NDCI were statistically significant, higher in Carignano regardless of treatment. The UV-B treatment reduced significantly the RWC only in Carignano leaves (**Supplementary Table S3**). In addition, TCARI/OSAVI and mARI indicated a lower relative abundance of chlorophylls and anthocyanins in Cannonau and a different leaf structure compared to Carignano. Moreover, the ND_LMA_ showed significant differences due to treatments, i.e., higher mass per leaf area in the +UV plants and slightly lower leaf dry matter in the −UV plants. At pea size (BBCH 73), the varietal differences were intensified, and the positive effect of treatments on NDCI increased in C and +UV compared to −UV, while TCARI/OSAVI increased in −UV plants, indicating chlorosis. Reflectance differences among treatments due to leaf structural characteristics were detected with ND_LMA_, the highest values being measured on C treatment and on Cannonau plants. At veraison (BBCH 83), the main differences in the vegetation indices were due to treatments, and TCARI/OSAVI was markedly higher in −UV plants of Cannonau and lower in the Carignano +UV and Cannonau C.

### 3.4 Differences in leaf anatomy and ultrastructure

**Figure 9:**
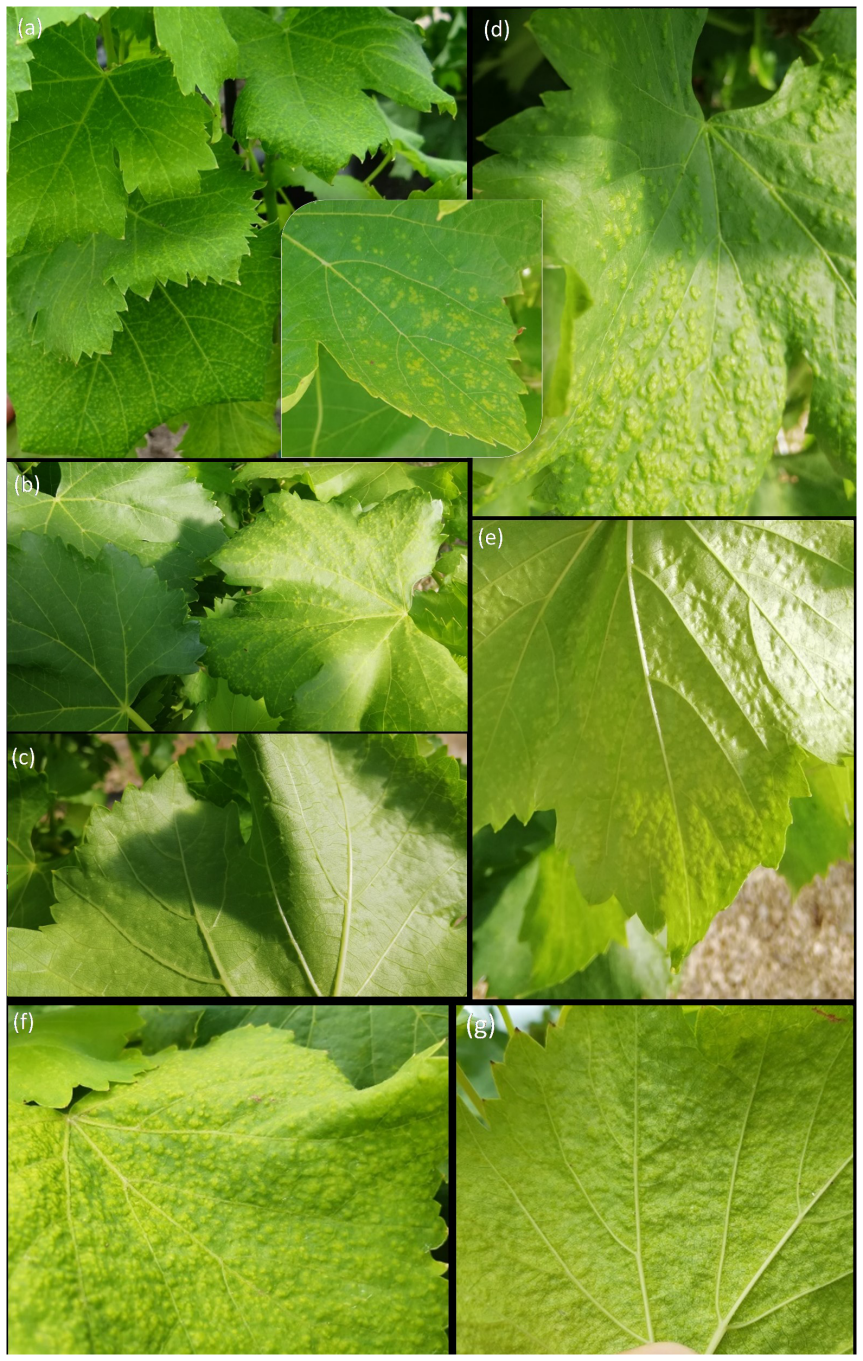
Leaf depigmentation spot and swelling formation and expansion on Cannonau −UV leaves inside the greenhouse. Depigmentation spots observed at the upper leaf blade during shoot growth [from BBCH 14–15; DOY 130) **(A)**; then, swelling symptoms started on the upper leaf blade, over the spots (BBCH 75; DOY 163) **(B)**; thereafter, lower-blade depressions caused by the swelling became visible (BBCH 77; DOY 177) **(C)**; finally, the swelling increased and expanded during the hottest months (BBCH 83; DOY 220) **(D, E)** until it reaches maximum volume by the end of the hot season (BBCH 89; DOY 255) **(F, G)**.

Beyond varietal ampelographic differences among Carignano and Cannonau leaves, the latter being much smaller, pale, and glabrous, inside the greenhouse, Cannonau plants and more conspicuously in −UV plants, the occurrence of leaf blade spot depigmentation was observed from early stages of shoot development (BBCH 14–15) and in all years of trial. The spots then evolved into depigmented, macroscopic swellings scattered on the leaf blade that expanded as the season advanced and *T*_air_ increased (**Figure 9**). This abnormality was most evident when the duration of very high temperatures reached or exceeded 10%, particularly in the −UV treatment. When the spot swelling symptoms were more pronounced on the leaf blade, the optical measurements of CHL, ANT, FLA, and NBI indicated absence of signal in many replications, indicating the transparency for the light flux of the leaf section sampled. These sensor’s reading failure indicated that chlorophyll excitation light pulses in the reference wavelengths and chlorophyll fluorescence light pulses for flavonoid estimation generated 100% transmission and zero fluorescence, respectively.

The areas of swelling, delimited and measured in the laboratory, on six adult leaves taken at veraison (BBCH 83) covered approximately 15.6% of leaf blades in Cannonau −UV and expanded further during ripening to ca. 25.6% of the leaf area by the end of ripening (BBCH 89) (**Supplementary Figure S6**). It was also possible to observe and delimit depigmentation and swelling on Cannonau +UV samples. However, in this case, the area affected was much circumscribed and reduced, covering approximately 2.3% and 4.2% of the single leaf area at veraison and at ripening, respectively. The C plants did not show such leaf blade deformations, and in Carignano this phenomenon was not seen. The aggravation of symptoms in Cannonau inside the greenhouse, as *T*_air_ increased up to 40°C, has led us to hypothesize that anatomical or ultrastructural differences within the leaf tissues caused by high *T*_canopy_ determined this phenomenon.

**Figure 10:**
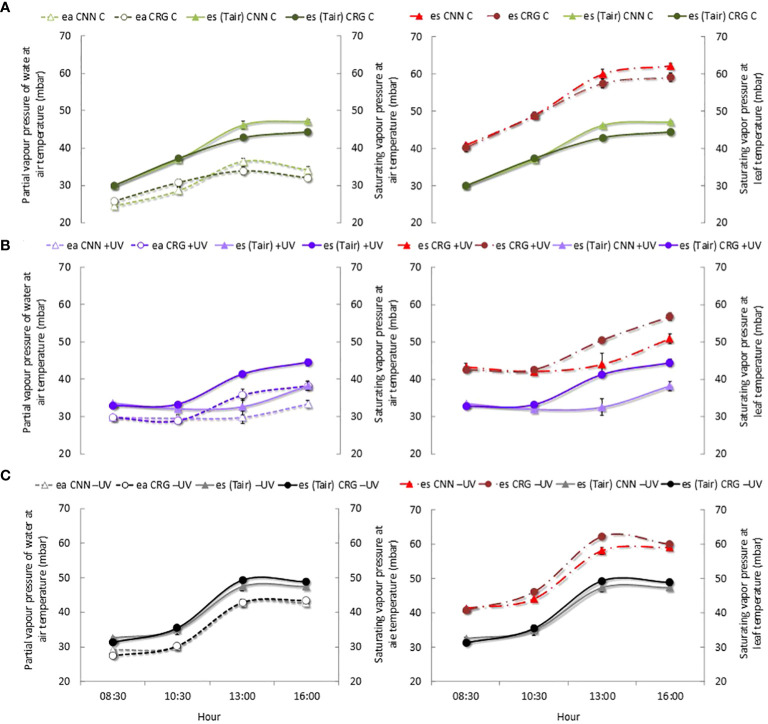
Patterns of actual vapor pressure (*e*_a_) and saturating vapor pressure (*e*_s_) at air temperature and saturation vapor pressure at leaf temperature (es *T*_leaf_) in Cannonau (CNN) and Carignano (CRG) plants subjected to C **(A)**, +UV **(B)**, and −UV **(C)** at veraison (BBCH 81) 2018. Mean values (*n* = 6) ± standard error.

**Figure 10** shows that at veraison (BBCH 83) the e_a_ in C plants varied among medium-low values during the day, much lower than the corresponding saturating vapor pressure, e_s_ (**Figure 10A**). Conversely, inside the greenhouse, the e_a_ values within the leaf mesophyll were higher and closer to the saturating pressure values (**Figures 10B, C**). In the −UV plants, e_s_ reached extremely high values and the e_a_ in the leaves was very close to those maxima (**Figure 10C**). Therefore, it is likely that the e_a_ within mesophyll interspaces and in almost empty cell protoplasm, in the depigmented areas of the leaves most affected by high temperatures, caused increasing swelling, leading to the deformation of the membranes. Besides tissue compression, the collapse of several mesophyll cells has certainly heightened this effect in Cannonau plants. The very rapid regression of the symptom of swelling as the *T*_air_ decreased and the leaves that were detached from the plant support the hypothesis that e_a_ at high temperatures caused the swelling of the depigmented and hollow leaf areas.

**Figure 11:**
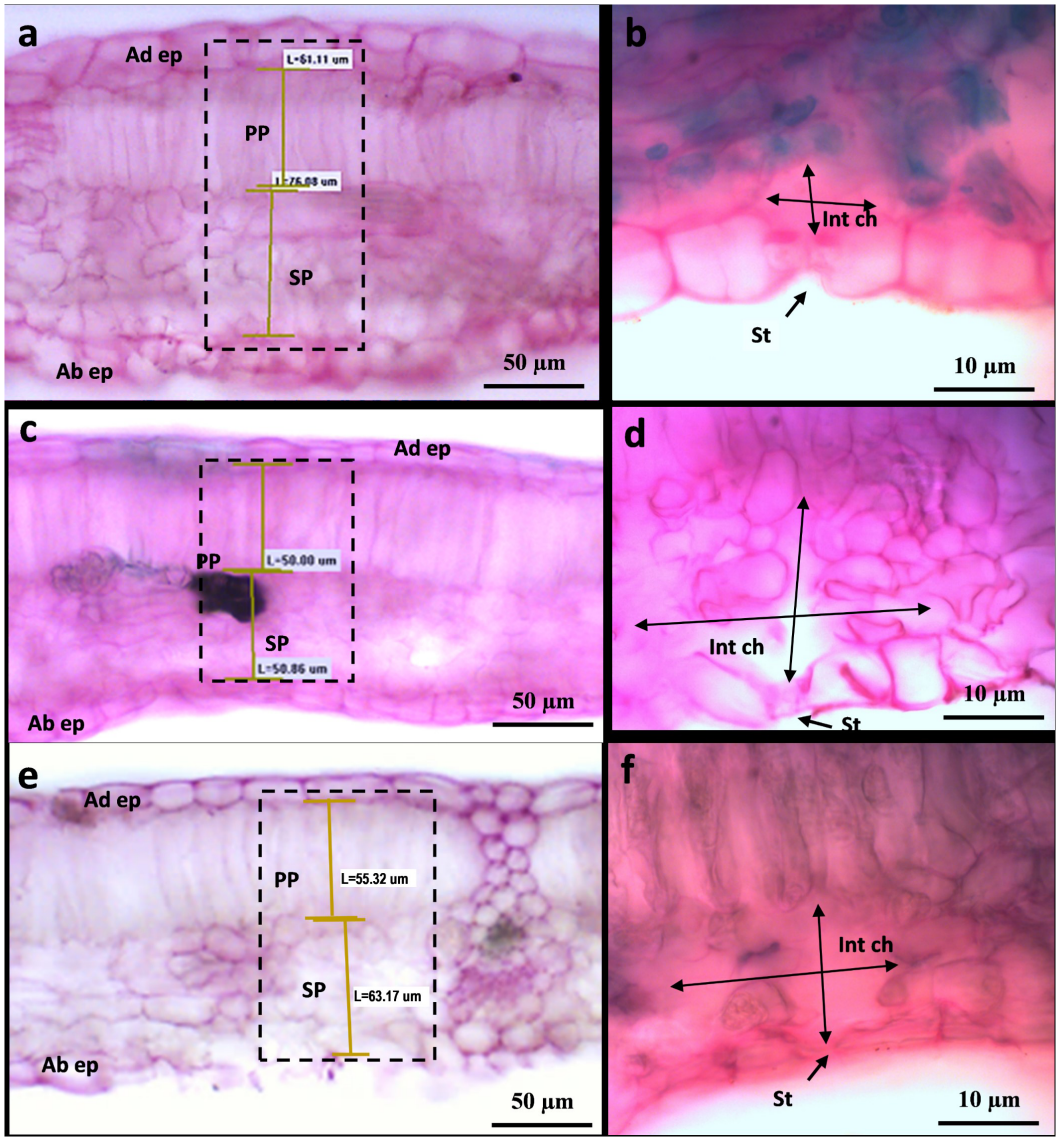
Leaf transverse sections of Carignano leaves (BBCH 83) under control condition I **(A, B)**, under heat and UV-B light (+UV) **(C, D)**, and under heat and absent UV light (−UV) **(E, F)** observed by photonic microscopy at ×20 **(A, C, E)** and ×100 **(B, D, F)**. The photograms in A, C, and E show leaf sections observed at ×20, while B, D, and F show details of leaf abaxial epidermis (Ad ep), palisade (PP) and spongy (SP) parenchyma, stomata (St), intercellular chamber (Int ch), and adaxial epidermis (Ab).

**Figure 12:**
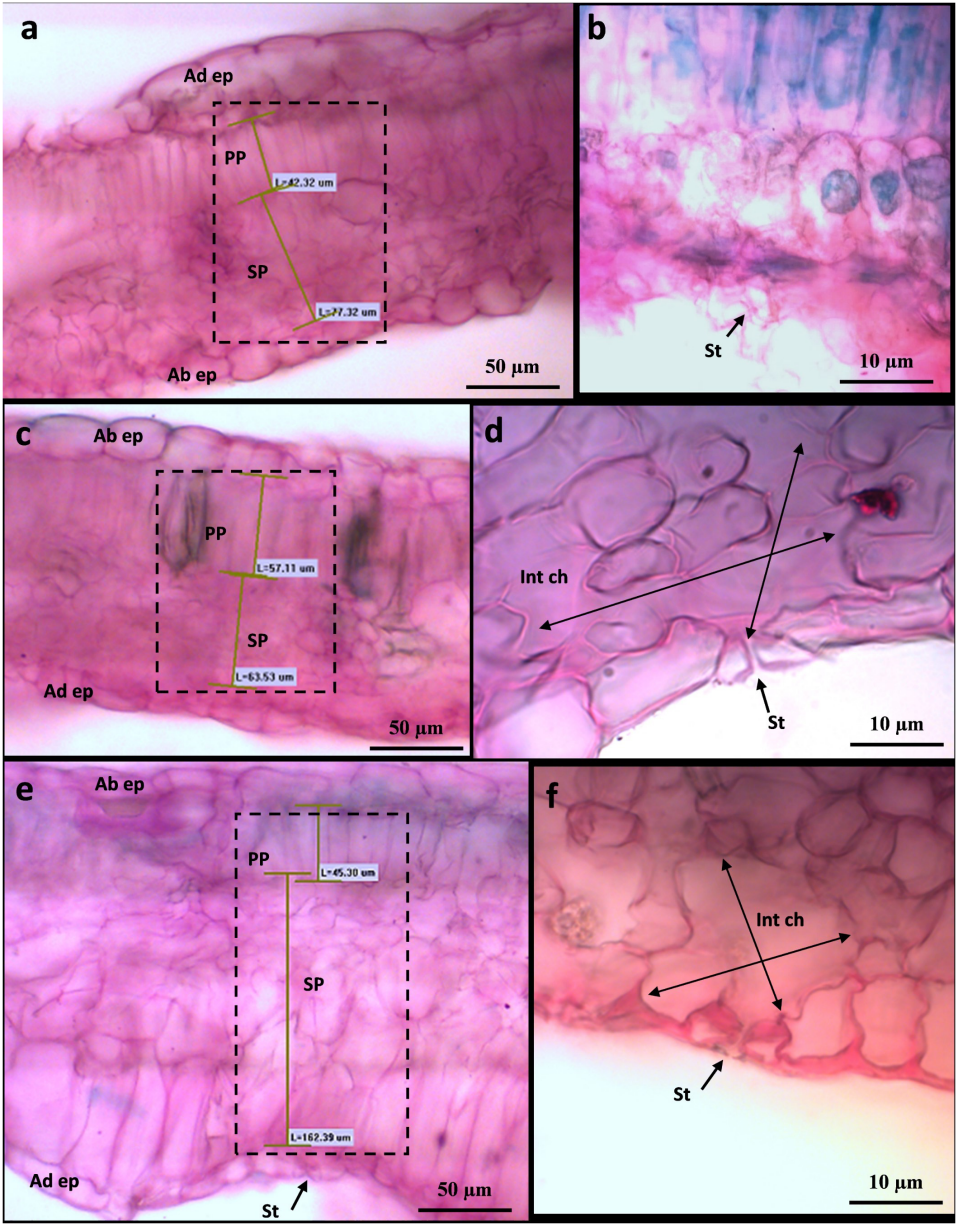
Leaf transverse section of Cannonau leaves (BBCH 83) under control conditions **(A, B)**, under heat and UV-B light (+UV) **(C, D)**, and under heat and absent UV light (−UV) **(E, F)** observed by photonic microscopy at ×20 **(A, C, E)** and ×100 **(B, D, F)**.

**Figure 13:**
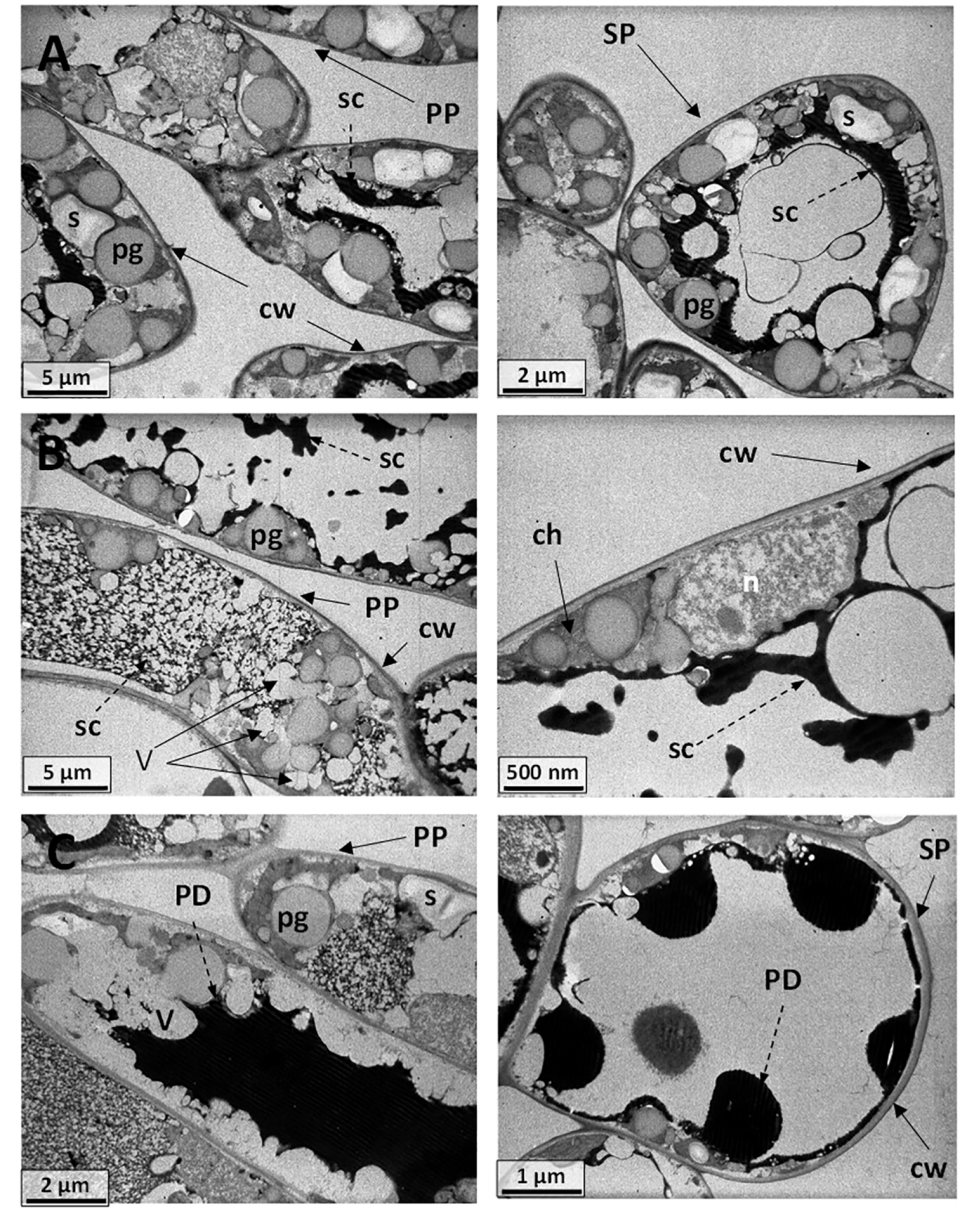
**(A)** Electromicrograph of cell ultrastructure of cross-sections through the leaf blades of **(A)** Cannonau control plants (BBCH 83) showing palisade (PP) and spongy (SP) parenchyma cells, with regular intercellular spaces and cell walls (cw), cytoplasm, and organelle integrity. Nucleus (n), plastoglobules (pg), central vacuoles (CV), membrane vesicles (V), starch grains (s), secretions (sc), electron-dense droplets (PD), probably phenolic deposits. **(B)** Cannonau −UV leaf specimens evidencing vacuolation of the cytoplasm, membrane dilation, with electron-dense flocculent content (sc) in the central vacuole (CV), swollen chloroplasts (ch), plastoglobules (pg), and starch grains (s). **(C)** Cannonau +UV leaf sections showing cell vacuolation of the cytoplasm and membrane dilation but less disturbance of the organelles, nucleus (n), plastoglobules (pg), vesicular phenolic deposits (PD) in spongy mesophyll cells, ultra-dense deposits (PD) fully filling the cell central space of palisade cell (dotted arrows), and a large series of ultra-dense droplets (PD) inside the central vacuole (CV) of the spongy mesophyll cells.

To shed more light on the causes of such leaf shape alteration affecting Cannonau −UV and, albeit to a lesser extent in Cannonau +UV, the anatomical differences between the two varieties and the three treatments were further investigated in the laboratory, on samples collected at veraison (BBCH 83) and mid-ripening stages (BBCH 85), by observing cross-sections of leaf blade on photonic microscope (**Figures 11**, **12**; **Supplementary Figure S7**) and also the cell ultrastructure of cross-sections through leaf blades on a transmission electron microscope (**Figures 13** and Cameron et al., 2022). Both varieties had a single layer of palisade parenchyma and spongy parenchyma. The main anatomical differences between treatments regarded leaf thickness, proportions of epidermis, spongy and palisade mesophylls to the whole leaf thickness, and, finally, the size of intercellular spaces *versus* thickening of lacunose parenchyma. In Carignano C, parenchyma thickness was higher than in the greenhouse treatments. However, the overall proportion of these tissues to the whole leaf thickness was lower because the epidermis was also much thicker (**Figures 11A**, **12A**; **Supplementary Table S6**). In the +UV treatment, spongy parenchyma thickness was much lower, and in Cannonau −UV a marked increase in thickness and densification of the spongy mesophyll cells adjacent to the adaxial epidermis was observed (**Figure 12F**). The differences between varieties under −UV treatment mainly concerned unevenness of leaf section and high thickness and cell density of spongy mesophyll in the cross sections of Cannonau leaves compared to Carignano (**Figures 11**, **12**; **Supplementary Figure S7**). This was due to both the formation of swelling areas on the leaf blade and the compression of the tissues in the adjacent areas under high temperature and leaf e_a_ in Cannonau −UV plants.

**Figure 14:**
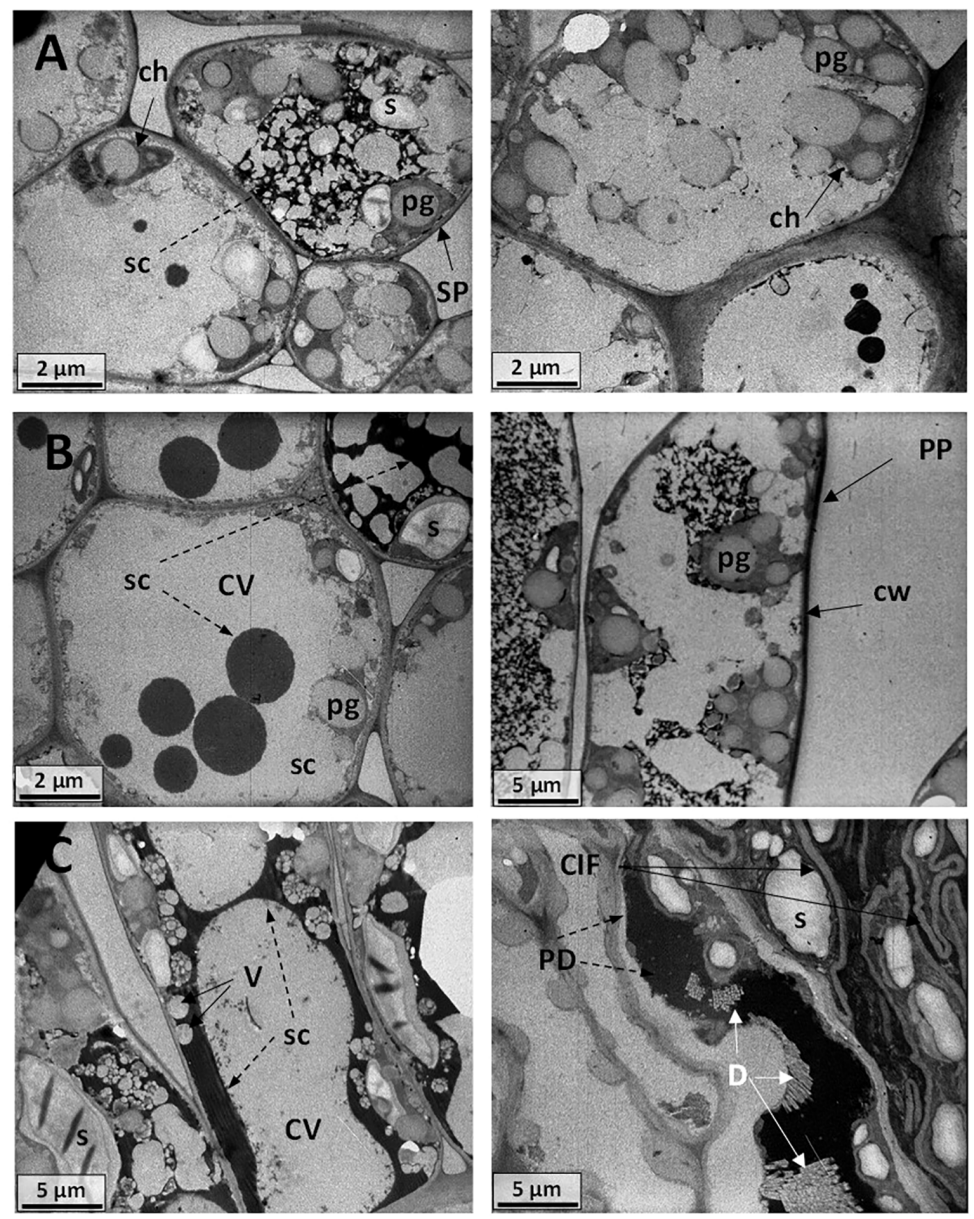
Electromicrograph of cell ultrastructure of cross-sections through the leaf blades of **(A)** Carignano control plants (BBCH 83) with regular cell walls (cw), central vacuoles (CV), swollen chloroplasts (ch), plastoglobules (pg), and secretions (sc). **(B)** Carignano −UV leaf cells showing swollen chloroplasts (ch), plastoglobules (pg), and different secretions (sc). **(C)** Carignano +UV plants with thick cell wall (cw), large plastoglobules (pg), convoluted invaginations and folding (CIF) of the cell wall and plasma membrane, starch grains (s), electron-dense deposits (PD), raphides, and druses crystals **(D)**.

Also ultrastructural differences were observed by transmission electron microscopy on leaf blade cross sections (**Figures 13**, **14**). The electron micrographs of Cannonau C specimens showed a number of large plastoglobules within the thylakoid membrane, along with large central vacuoles water filled (**Figure 13A**). There were multiple starch grains and different electron-dense material lining the organelles in the cytoplasm. Cannonau –UV leaf sections showed cells with cytoplasm vacuolation, and electron-dense flocculent content with fluid aspect associated with chloroplast or nucleus envelopes, or connected with groups of small, electron-empty vesicle in the central vacuole (**Figure 13B**). Numerous plastoglobules were observed in the thylakoid membranes. As compared to −UV, the +UV leaf cells showed similar vacuolation of the cytoplasm and membrane dilation, but less organelle disturbance (**Figure 13C**), although chloroplast swollen and central hyaline vacuole expansion were evident. Plastoglobules, massive electron-dense deposits, probably of phenolic compounds, were observable, either as vesicular deposits aside the organelles in the spongy mesophyll cells, or fully filling the cell central space, or forming large series of ultra-dense droplets inside the central vacuole the mesophyll.

Leaf ultrastructure in Carignano C plants also showed extensive vacuolization with both large central vacuoles and vesicles (**Figure 14A**). Swollen chloroplasts with a number of plastoglobules of great size were observed and electro-dense deposits were visible. The −UV Carignano leaves cells showed secretory deposition and large cell vacuolation (**Figure 14B**). The micrographs of Carignano +UV leaves showed quite intense cell membrane activity (**Figure 14C**). Besides to the thick cell wall, swollen chloroplasts and large plastoglobules, also convoluted invaginations and folding of the cell wall and plasma membrane were observed. These structures formed a complex net in the inner cell walls, enveloping numerous starch grains and electro-dense deposits. The +UV Carignano leaves also presented a number of raphide and druses crystals.

## 4 Discussion

Bud break and flowering were anticipated and slightly shortened in the greenhouse as compared to the control. The Normal Heat Hours index reflected quite well the influence of temperatures on vine physiological performance, and it represented closely the timing of phenological succession, particularly flowering. The stages most sensitive to heat stress were bud break, flowering, berry growth and ripening stages (Jones et al., 2005; Duchêne et al., 2010; Greer et al., 2010; Bernardo et al., 2018). In recent decades, there has been a shortening of the various stages of grapevine growing cycle in many worlds’ wine-growing regions, as a result of global warming (Hannah et al., 2013; Dinu et al., 2021). Besides to phenological interval shortening and advances in vegetative restart (Alikadic et al., 2019; Cameron et al., 2022), the persistence of high temperatures during fruit growth and ripening, can severely limit photosynthetic activity, slowing translocation of photosynthates and fruit ripening (Greer and Weedon, 2013). Phenological advances in early growth, markedly affect fruit formation, increasing the risk of exposing young shoots to spring frost (Poni et al., 2023). However, in natural environments, phenological advances caused by transient heat, shift later phenological stages to cooler conditions, and this tends to dampen the effect of the temporary warming that previously occurred. Moreover, an increased celerity of phenological stages, driven by high temperature, still depends upon the plant source/sink balance and photosynthates availability to support further plant development (Sadras and Moran, 2013). Nevertheless, when the persistence of elevated temperatures during flowering and ripening is high, fruit set and ripening metabolism can be severely limited (Gouot et al., 2019; Ryu et al., 2020).

In our study, the *T*_canopy_ of C plants was lower than that of the +UV and −UV plants for the entire duration of the trial, and in summer the greenhouse *T*_air_ exceeded 35°C for more than 1/3 of the heat stress duration, while in the outdoor environment it remained within 6% of the recorded *T*_air_. In the hottest months, the duration of *T*_air_ > 40°C on canopies was very high in the greenhouse, varying between 11 and 18%, while in the outdoor environment the permanence of this threshold remained below 1%. During daytime, *T*_air_ – *T*_canopy_ was significantly higher in Cannonau +UV plants, followed by −UV and then C of both varieties. Carignano C showed sharper decrease during the morning, while Cannonau C maintained quite constant canopy temperature depression along the day (**Supplementary Figure S4**).

Cannonau plants had lower transpiration rate during the afternoon than Carignano, although the differences between varieties were not statistically significant in −UV. The differences can be attributed to the lower stomatal conductance in Cannonau, yet the crucial role of the environment on the degree of iso-hydric behavior displayed by the cultivar is also confirmed (Lovisolo et al., 2016; Hochberg et al., 2018). The greater stomatal closure in Cannonau leaves in C environment, for which intrinsic water use efficiency was significantly higher than that of Carignano and the smaller differences in stomatal closure between varieties inside the greenhouse, under water comfort conditions, confirms that the near-isohydric behavior is strongly affected by the environment, particularly when soil water availability is not a limiting factor (Tramontini et al., 2014; Feng et al., 2019b). Despite the exposure to heat stress for a long period of time, the water comfort and efficient use of light allowed these plants to reach a higher photosynthetic rate and primary photochemical activity than those in the outdoor control, resulting in a more intense vegetative development. In the greenhouse, diffuse PAR and early morning compensation with PAR lamps have probably helped in optimizing PSII adjustment, particularly before the heat stress became severe (Greer and Weedon, 2013). A strong increase in shoot elongation and leaf wall growth was observed in both varieties. However, enhanced leaf area per plant was achieved by the emission of a higher number of leaves per shoot in Cannonau, while in Carignano an important part of the substantial difference was due to single leaf area increases (Fernandes de Oliveira et al., 2019). The resource investment on leaf development, with continuous increases in single leaf area under the high *T*_air_ in the greenhouse, was observed in Carignano +UV even after veraison, highlighting the UV-B stimulation effect on leaf metabolism in plants under stress conditions (Berli et al., 2011, 2013; Martínez-Lüscher et al., 2015; 2016b).

The two grapevine varieties exhibited different plasticity in adapting leaf gas exchange and vegetative growth to light and heat stress conditions. The tighter stomatal control of Cannonau and reduced soil water availability, which markedly limited transpiration under control conditions, could partly explain the different persistence of high *T*_canopy_ observed between varieties under the −UV treatment. However, similar varietal differences were not found in +UV plants, which were also subjected to long-term high temperatures.

In addition, the balance of photosynthetic and non-photosynthetic pigments also differed across treatments and varieties. In fact, the correlation patterns between estimated and measured leaf pigment and nitrogen contents highlight the catalytic effect of UV radiation on anthocyanin and flavonol synthesis in grapevine leaves (Martínez-Lüscher et al., 2014) over the growing cycle. Besides this, the high permanence of very high temperatures during berry development and ripening (BBCH 75–89) negatively affected the ability for anthocyanin synthesis and accumulation in leaves, more so in Cannonau, which is known to be more sensitive to anthocyanin degradation under heat stress (Mori et al., 2007; Fernandes de Oliveira et al., 2015). Under the UV-B treatment, the sensitivity to heat of Cannonau seemed to have been partly compensated.

Leaf phenolic estimation in Carignano and Cannonau suggests that different levels of antioxidant metabolite synthesis and different metabolic balance occurred in the two varieties in response to changes in light and thermal environment, which allowed them to adapt differently to the abiotic stimuli to ensure homeostasis (Carvalho et al., 2015b) and physiological performance (Fernandes de Oliveira et al., 2019). The fluctuating behavior of pigment indexes along the season can be explained by both a reduced traceability, namely of flavonols, under high temperatures and the chloroplast avoidance movement in response to increasing *T*_leaf_, which reversibly realigns chloroplasts on anticlinal cell surfaces, in orthogonal position to the leaf surface, in order to minimize the absorption of photons and excess radiant energy (Nauš et al., 2010). Nevertheless, the response of leaf FLA index to heat and UV-B radiation is an important issue to be considered when evaluating the shielding defense role of these compounds against excessive radiation and biotic agents (Agati et al., 2013; Martínez-Lüscher et al., 2014; Daryanavard et al., 2023; Gautam et al., 2023). In red grape berries, the UV-shielding role is carried out mainly by high concentrations of anthocyanins, while the amounts of flavonols are much smaller and less reactive and sensitive to changes in UV light or *T*_canopy_ above 35°C (Mori et al., 2007; Martínez-Lüscher et al., 2016a). Yet during the stages of intense vegetative growth, high shoot hydraulic conductance, and daily leaf gas exchange, leaf phenolic contents are very low. However, in leaves exposed to high levels of UV-B radiation, a rapid reaction of flavonol synthesis can partially compensate for the lack of anthocyanin accumulation caused by heat stress, thus allowing efficient tissue protection (Aphalo et al., 2012; Daryanavard et al., 2023). In the absence of UV-shielding pigments, excessive radiation leads to a reduction in primary photochemical efficiency and photosynthesis, and increased heat dissipated with a higher frequency of photoinhibition and photooxidation phenomena (Osmond and Chow, 1988). In this study, despite the variation in pigment indexes observed in Carignano, the stimulating effect of UV-B radiation on photosynthetic pigments in both varieties seemed evident when comparing greenhouse treatments with the control. In addition, the estimated phenolic contents suggest that UV-B was particularly effective inside the greenhouse and in Carignano. These may justify a lower need for further antioxidant pigments in leaf tissues. Different sensitivity and levels of tolerance to abiotic stressors have been found among grapevine varieties, including basal levels of antioxidant metabolite synthesis, which depends on the genetic plasticity in antioxidant homeostasis (Carvalho et al., 2015a).

Furthermore, the significant differences in normalized reflectance spectra between varieties and among treatments also confirmed a different balance between photosynthetic and non-photosynthetic pigments in the two varieties and indicated higher pigment content in +UV leaves, suggesting also a different evolution of leaf structure in response to treatments in Cannonau and Carignano (Gitelson and Merzlyak, 1994; Daughtry et al., 2000; Merzlyak et al., 2003). The differences in reflectance for wavelengths greater than 1,300 nm, which are inversely proportional to the amount of water content present in the plant, can be dictated by differences in leaf thickness (**Supplementary Table S4**) (Ng et al., 2007; Cheng et al., 2014). Importantly, the different relationships observed between estimated indices and actual metabolite content also suggest that the high performance in photochemical and photosynthetic activities in both varieties seemed to be achieved by a different metabolism and quantitative balance of metabolites with ROS scavenging capacity and that the mechanisms of antioxidant homeostasis were activated differently in the two varieties (Carvalho et al., 2015b). Moreover, in addition to metabolic factors, leaf anatomical and ultrastructural differences may largely explain the observed spectral differences. In this regard, spot depigmentation and swelling in the leaf blades of Cannonau plants inside the greenhouse, widespread under −UV treatment and expanded during summer, highlight a strong impact of heat stress, observed previously on other species (Buchner et al., 2007; Grigorova et al., 2012). The sensitivity of Cannonau to heat stress, in terms of anthocyanin degradation and stomatal closure compared to Carignano (Fernandes de Oliveira et al., 2015; 2019), may partly explain why these symptoms affected only Cannonau. From an anatomical point of view, the rare presence of trichomes, only in the lower leaf blade, has probably contributed to the lower protection of leaf tissues against excessive light damages (Zhang et al., 2021), followed by spot depigmentation and swelling, right from the beginning of the leaf growth cycle. Conversely, in Carignano, the bristly trichomes in both upper and lower layers likely helped in supporting leaf mechanical resistance during the prolonged abiotic stress inside the greenhouse (Nieddu, 2011; Wang et al., 2021; Amada et al., 2023).

The lower mesophyll thickness in greenhouse plants observed in Carignano is likely due to the daily reduction in direct light exposure inside the greenhouse (Yun and Taylor, 1986; Smith et al., 1998; Feng et al., 2019a). The shadow effect is typically characterized by lower leaf blade thickness, increased chlorophyll content per reaction center, and higher Chl b/Chl a and PSII/PSI ratios. In fact, a typical anatomical adaptation response to heat stress would be an increase in leaf blade thickness and in the proportion of spongy parenchyma (Wahid et al., 2007; Salem-Fnayou et al., 2011). However, though having lower epidermal and leaf thickness, the greenhouse plants had much wider intercellular spaces of spongy parenchyma than in C plants (**Figures 11D–F**, **12E–G**). This latter morpho-anatomical differentiation between outdoor and greenhouse plants can be attributed to high water content and leaf e_a_ under high soil water availability and low air VPD inside the greenhouse (Amitrano et al., 2021).

At the whole canopy level, leaf and shoot morpho-anatomical differences as those observed among these two varieties may influence heat stress responses—for instance, the higher single main leaf area, presence of trichome, and low secondary leaf area growth of Carignano favor plant heat loss, net assimilation, and source–sink balance during berry growth and repining. Conversely, the higher presence of young lateral shoots contrasts fruit growth and ripening and increases daily plant water use (Douthe et al., 2018; Poni et al., 2023). In fact, low fruit set and severe early fruit dropping occur in this variety (Nieddu, 2011) with high bud load and light or delayed summer shoot pruning. At the leaf scale, the increased intercellular spaces of spongy mesophyll of Cannonau plants exposed to heat stress and −UV have probably limited, to a further extent, leaf hydraulic and stomatal conductance in Cannonau, leading to higher *T*_leaf_ and heat stress duration, yet the role of the environmental conditions on plant hydraulic behavior may be as significant as the cultivar in determining water potential and stomatal control over transpiration (Hochberg et al., 2018; Gambetta et al., 2020).

In this study, the differences in stomatal conductance and transpiration rate during the afternoon lead to significant differences in *T*_air_ – *T*_canopy_ among treatments and varieties during the day, with higher values in Cannonau +UV and almost constant *T*_air_ – *T*_canopy_ difference under C conditions, particularly in Cannonau. Moreover, inside a greenhouse, a higher spongy mesophyll porosity helps plants to increase intercellular aeration to extend the internal CO_2_-absorbing surface, together with mesophyll conductance and gas exchange. Such adaptation, triggered under a hot environment and in the absence of consistent aerodynamic air and water vapor transport from the leaf surface to the surrounding atmosphere, also contributes for improving photosynthetic response (Huang et al., 2022; Wohlfahrt et al., 2022).

Leaf spot swelling observed in −UV Cannonau plants determined the thickening of leaf tissues (**Figure 12**; **Supplementary Figure S7**), more evident in the areas where cell wall elasticity was higher and the density of protoplasts of tissues nonspecialized in water conduction was lower (Aasamaa et al., 2005). In previous studies concerning the anatomical and physiological characteristics of *Populus tremula* leaves during their ontogeny, Aasamaa et al. (2005) pointed out that protoplast characteristics may be more important in determining differences in leaf hydraulic conductivity between leaves grown under different environmental conditions than differences in the characteristics of xylem or parenchyma cells. Confirming this hypothesis, a number of differences in leaf tissue ultrastructure, protoplast density, as well as cell metabolic activity were found between varieties and among treatments in our study, when observing the cross-sections through the leaf blade with the transmission electron microscope.

The uniformity and integrity of cells and protoplasts in the leaf specimens of Cannonau C showed palisade and spongy parenchyma with cell walls of regular structure and ample intercellular space as well as cytoplasm, organelles, and nucleus contents that were well organized and intact. The number of starch granules indicates intense photosynthetic metabolism, starch accumulation in plastids or specialized amyloplasts, typically found on adult leaves in good vegetative condition (Salem-Fnayou et al., 2011). However, the number and size of plastoglobules involved in biosynthetic pathways such as that of tocopherols, which protect membranes from reactive oxygen species (ROS), and the presence of electron-dense deposits in the vacuole, around the cell organelle, suggests intense cellular activity under the heat stress conditions of the site (Zavaleta-Mancera et al., 1999; Salem-Fnayou et al., 2011; Shanmugabalaji et al., 2022). The globular shape of the cells and vacuoles indicate high cell turgor as also did the midday stem water potential above −0.6 MPa measured at veraison (BBCH 81), while the lower RWC in Carignano +UV was related to the higher leaf mass (**Supplementary Table S3**). The vacuolation of cells, the presence of smaller vesicles, and the accumulation of secretory deposits indicate the onset of leaf senescence (Castro and Demarco, 2008; Costa et al., 2013; Ribeiro et al., 2021). The vesicles are lytic vacuoles, known as senescence-associated vacuoles, that play a key role in chloroplast proteolysis and amino acid and peptide release to be reallocated in other plant organs (Costa et al., 2013). Also in Cannonau leaf blades subjected to heat and −UV, the electron micrographs showed cells with evident vacuolation of the cytoplasm (Youssef et al., 2018), with membrane dilation and swollen chloroplasts. Moreover, the lower number of starch grains, the number and size of plastoglobules within chloroplasts, and the accumulation of electron-dense flocculent deposits (possibly oil droplets, tannins, and other phenolic compounds) in central vacuoles also indicate that intense enzymatic activity was triggered (Musetti et al., 2007; Grigorova et al., 2012; Hu et al., 2020). The numerous plastids, plastoglobules, and the fluid aspect of the dark deposits around the thylakoid membranes and nucleus envelopes and electron-empty vesicles also suggest that the treatment induced intense oxidative stress (Austin et al., 2006; Costa et al., 2013; Golczyk et al., 2014; Rottet et al., 2015; Carvalho et al., 2015a). Compared to −UV, the +UV Cannonau leaf cells showed similar vacuolation of the cytoplasm and membrane dilation, but less disturbance of the organelles. Taking into consideration that the Cannonau +UV leaves were subjected to similar *T*_air_ ranges and duration as those under –UV treatment, the morphological and ultra-structure observation also suggest a higher capacity of +UV plants of triggering more efficient heat dissipation mechanisms and reducing ROS formation (Zhang et al., 2018). The UV-B treatment seemed to have promoted higher synthesis and accumulation of phenolics in leaves crucial for enhancing the ROS scavenging capability (**Figures 7**, **13**, **14**). The influence of cultivar and treatment on leaf phenolic accumulation was statistically significant during flowering (BBCH 65) and from veraison until leaf senescence (BBCH 83–92) (**Supplementary Table S4**), confirming different activation of phenolic synthesis and an effective antioxidant potential of the UV-B dose in leaves, though with small phenolic contents (Agati et al., 2013). No direct effects of UV radiation on stomatal closure and *T*_leaf_ were detected as reported in other studies (Williams et al., 2022). This could be explained by the major role played by high temperatures (>40°C) and high permanence of heat stress on stomatal control and oxidative damage. The non-limiting water availability could also have reduced the magnitude of a possible stomatal response to +UV treatment (Tossi et al., 2009; Williams et al., 2022).

As compared to Cannonau C, the C leaves of Carignano showed denser mesophyll, thicker cell walls, and narrow intercellular spaces (**Figures 11**–**14**). Yet the large central vacuoles and vesicles indicate intense cell vacuolization similarly as that observed in Cannonau C. Compared to −UV Cannonau, in the −UV Carignano leaves specimens less membrane dilation were observed. In the Carignano leaves subjected to both heat and UV-B radiation, further mechanisms of stress response regulation were evidenced (Carvalho et al., 2015b). Besides the different secretions, namely phenolic deposition, other membrane repair mechanisms usually involved in cell protection from biotic and abiotic stresses were observed (Musetti et al., 2007; Castro and Demarco, 2008). Along with mesophyll-extensive electron-dense deposits, many druses and raphide crystal were also observed. These calcium oxalate crystals can have different functions, from mechanical support for stiffening leaf tissues (Nakata, 2003) to ion regulation and photosynthesis optimization by light gathering and scattering, or even as carbon reservoir, under stomatal closure, that can be decomposed to CO_2_ by oxalate oxidase (Lawrie et al., 2023). The UV-B light dose applied to the Carignano plants seemed to have activated other membrane and cell wall barriers, including convoluted invaginations of the plasma membrane (Ljubešić and Britvec, 2006; Wang et al., 2022), forming a net in the inner cell wall around numerous and large starch grains and secretions. Previous studies of Tripathi et al. (2019) reported alteration in the balance of cell proliferations, differentiation, intercellular communication, and morphogenesis in sunflower leaves under supplemental UV-B, and the ultrastructure alterations were emphasized by the combination of two abiotic stresses—UV-B + ozone, and in that study, differential cell growth was among the underlying reasons for such alterations (Tripathi et al., 2019). Cell wall folding has also been identified as an adaptive mechanism to prevent cell death in plant with vegetative desiccation tolerance under drought (Liu et al., 2024). In our study, the combination of UV-B light with heat stress seemed to have allowed the Carignano plants triggering different metabolic pathways to provide leaf tissue protection. The UV-B light promoted the accumulation of ROS scavenging compounds instead in Cannonau plants to a larger extent compared to C and –UV plants, but the production of calcium oxalate crystals or membrane invagination was not observed.

In conclusion, a divergent adaptive evolution of these two traditional Sardinian grapevine varieties led to quite distant genotypes and phenotypes, including leaf anatomical, ultrastructural, and physiological abilities to cope with prolonged heat and high solar radiation. Besides the genetic distance among varieties, the acclimation to hot and dry environments like those where Carignano is usually cultivated in Sardinia may have also promoted the higher activation of more effective antioxidant defense responses. The diverse phenotypic plasticity of grapevine varieties response to abiotic stresses is achieved by different metabolic pathways and/or signaling activation levels (Venios et al., 2020). In some cases, a highly efficient heat stress tolerance strategy may involve large morpho-anatomical changes; thus, further research work is needed to better understand the role of leaf anatomy and morphological structure of vines in their adaptation to environmental stresses. In the study area, Carignano proved to be well acclimated to very hot and dry environments due to the ability to keep high levels of different stress tolerance mechanisms and high yield and wine quality (Fernandes de Oliveira and Nieddu, 2016b). Meanwhile, Cannonau seems well adapted to mountain areas due to the positive effect of higher UV-B intensity and thermal excursion on cultivar heat stress tolerance, its high water use efficiency and physiological performance under water deficit, and the ability to reach better anthocyanin and phenolic profile in berry and wine at high altitude (Mercenaro et al., 2019).

In order to better address the challenges that global warming poses to viticulture and support grapevine breeding programs and growers’ decisions in each territory, especially in climate change hotspots (Cos et al., 2022), it is important to characterize varietal differences in primary and secondary metabolism involved on abiotic stress tolerance, focusing on cultivar or clone morpho-anatomical traits to cope with sub-optimal growing conditions, the range of defense mechanism activated, and the efficiency of phenotypic responses evolving under different environments. Further research is needed concerning the interaction effect of vineyard altitude × cultivar to evaluate the extent of positive effects of high daily UV-B doses and duration of high temperatures with increasing altitudes. This would help in better addressing future potential shifts in vineyard cultivation toward different altitudes in order to keep vineyard performances and fruit quality high with suitable varietal and sustainable vineyard management, particularly in global warming hotspots of Mediterranean-climate wine-growing areas.

## Data availability statement

The raw data supporting the conclusions of this article will be made available by the authors, without undue reservation.

## Author contributions

AFO: Conceptualization, Data curation, Formal analysis, Investigation, Methodology, Writing – original draft, Writing – review & editing. GKP: Data curation, Formal analysis, Writing – original draft. SN: Data curation, Formal analysis, Writing – original draft. GB: Data curation, Formal analysis, Methodology, Writing – original draft. SM: Data curation, Formal analysis, Methodology, Writing – review & editing. MR: Data curation, Formal analysis, Methodology, Writing – review & editing. DS: Supervision, Writing – review & editing. SB: Methodology, Supervision, Writing – review & editing. GN: Methodology, Supervision, Writing – review & editing.
