# Supplementary material for: UV light and adaptive divergence of leaf physiology, anatomy, and ultrastructure drive heat stress tolerance in genetically distant grapevines
Source: Front Plant Sci. 2024 Jun 18;15:1399840. doi: 10.3389/fpls.2024.1399840 (PMC11217527; doi:10.3389/fpls.2024.1399840)
Supplement: Supplementary file 1 [file DataSheet_1.docx]

Supplementary Material

# Supplementary Data

Patterns of global solar radiation (Rg) and air temperature (T_AIR_) in three replicates (r1, r2 and r3) of control plants (C), during the months of June, July, August and September 2019.

Patterns of global solar radiation (Rg) and air temperature (T_AIR_) in three replicates (r1, r2 and r3) of +UV plants inside the greenhouse during the months of June, July, August and September 2019.

Patterns of ultraviolet B radiation (UV-B) and air temperature (T_AIR_) in three replicates (r1, r2 and r3) of control plants (C) during the months of June, July, August and September 2019.

# Supplementary Figures and Tables

## Supplementary Figures

**Supplementary Figure 1.** Correlation models of observed (single sensors and spectrosense+, Llandrindod, Wells, UK) and estimated (single sensor GMR instruments, FI, IT) data, for continuous recording, of intercepted global solar radiation (Rg) and photosynthetically active radiation (PAR) intensity.

**Supplementary Figure 2.** Hourly data of global solar radiation (Rg), PAR, UV-A and UV-B incident at the experimental site, in the control (left), and transmitted by the UV-screening panels inside the greenhouse (right), during a clear sunny day of June 2018

## Supplementary Figure 3. (A) Percentages of Rg and UV-B intercepted throughout the season within the canopies (at 60 cm from the shoot apex) in control (C) and +UV plants, recorded at the vegetative apex (at 10 cm from the shoot apex) and 60 cm below; (B) Average daily trend of Rg and UV-B (lines) and average accumulated intensity (histograms) in control and +UV canopies, at an average canopy height (60 cm apex distance) (b); (C) Solar irradiance transmitted inside the greenhouse and into the outdoor environment, measured in June, on a clear day, at mid-morning (10.00 h) and in the afternoon (15.00 h), outside and inside the greenhouse.

##

## Supplementary Figure 4. Mean patterns of average season relative humidity (RH) and air temperature (T_air_) and differences between air and canopy temperature in Carignano (CRG) and Cannonau (CNN) plants subjects to the treatments C, ‒UV and +UV from Bud break until leaf senescence (BBCH 9 – 93).

## Supplementary Figure 5. (A) Patterns of single leaf area, from flowering until the beginning of leaf senescence; (B) pruning weight per plant; in Carignano (CRG) and Cannonau (CNN) plants subjected to C, +UV and –UV treatments in season 2019. Mean values (n=6) ± standard error.


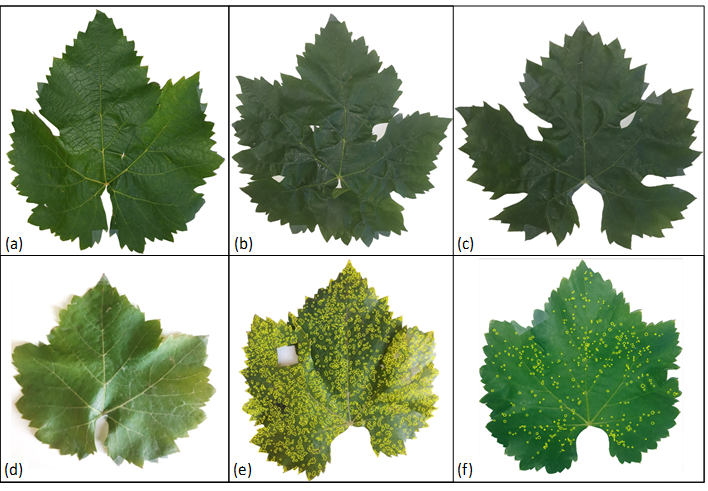


**Supplementary Figure 6.** Delineation of swelling areas in Carignano (a-c) and Cannonau (d-f) leaf samples subjected to C (a, d), –UV (b, e) and +UV (c, f) treatments, at veraison (BBCH 83) 2019.

**Supplementary Figure 7.** Transversal sections of Cannonau (a–c) and Carignano (d–f) leaves under control (a, d), ―UV (b, e), +UV (c, f) treatments, observed at veraison (BBCH 83) 2019 using optical microscope with ×2.5 objective.

## Supplementary Tables

**Supplementary Table 1.** Monthly average solar radiation, maximum (T max), mean (T mean) and minimum (T min) air temperature and precipitation of the 30-year reference period (1991-2010), and during the three study seasons 2017, 2018 and 2019. Data from ARPA Sardegna – Sardinia Regional Agency for Environmental Protection. Weather-Climatic Department (https://www.sar.sardegna.it/).

| Month | Year | Solar Irradiance  (W m^‒2^ day) | T max (°C) | T avg (°C) | T min (°C) | PP (mm) |
| --- | --- | --- | --- | --- | --- | --- |
| April | Climate normal | 790.2 | 19.1 | 13.9 | 8.8 | 51.0 |
|  | 2017 | 977.2 | 20.5 | 13.9 | 7.2 | 80.0 |
|  | 2018 | 969.2 | 22.3 | 15.9 | 9.8 | 56.8 |
|  | 2019 | 944.8 | 19.3 | 13.8 | 8.3 | 60.0 |
| May | Climate normal | 930.2 | 22.8 | 17.6 | 12.5 | 37.0 |
|  | 2017 | 980.5 | 25.6 | 18.6 | 11.2 | 12.0 |
|  | 2018 | 980.8 | 22.2 | 17.6 | 13.3 | 214.2 |
|  | 2019 | 1056.5 | 21.0 | 15.6 | 10.1 | 52.0 |
| June | Climate normal | 1289.7 | 26.7 | 21.6 | 16.4 | 18.0 |
|  | 2017 | 925.9 | 29.9 | 23.9 | 17.1 | 29.0 |
|  | 2018 | 1030.5 | 27.5 | 21.9 | 16.5 | 17.4 |
|  | 2019 | 934.4 | 30.4 | 23.9 | 18.3 | 1.8 |
| July | Climate normal | 1248.3 | 28.4 | 24.4 | 20.3 | 5.0 |
|  | 2017 | 909.2 | 32.1 | 25.7 | 18.8 | 11.0 |
|  | 2018 | 778.0 | 31.2 | 25.6 | 20.5 | 0.0 |
|  | 2019 | 898.1 | 31.8 | 26.1 | 20.7 | 2.4 |
| August | Climate normal | 994.7 | 28.9 | 24.9 | 20.8 | 6.0 |
|  | 2017 | 855.8 | 33.9 | 26.1 | 18.6 | 11.0 |
|  | 2018 | 905.6 | 30.8 | 24.9 | 20.1 | 45.4 |
|  | 2019 | 863.2 | 32.4 | 26.0 | 20.4 | 17.2 |
| September | Climate normal | 712.6 | 26.5 | 22.4 | 18.4 | 32.0 |
|  | 2017 | 861.1 | 26.4 | 20.7 | 15.1 | 114.0 |
|  | 2018 | 754.8 | 28.6 | 23.1 | 18.3 | 46.2 |
|  | 2019 | 812.7 | 28.8 | 23.1 | 18.1 | 34.8 |
| October | Climate normal | 503.4 | 22.8 | 18.7 | 14.7 | 73.0 |
|  | 2017 | 719.8 | 23.8 | 17.9 | 11.7 | 45.1 |
|  | 2018 | 658.5 | 24.4 | 19.2 | 15.1 | 115.6 |
|  | 2019 | 691.4 | 25.2 | 19.5 | 15.0 | 46.0 |

**Supplementary Table 2.** Chlorophyll (CHL), flavonol (FLA), anthocyanin (ANT) and nitrogen balance (NBI) indexes and total chlorophyll, chlorophyll b to chlorophyll a ratio, total anthocyanin and phenolics, nitrogen and water content of leaf samples. Average data in season 2018 and two-way ANOVA significance of differences of cultivar, treatment and interaction effects. Different uppercase letters indicate significant differences between cultivar and lowercase letters indicate significant differences among treatments.

| cv | Treatment | CHL | FLA | ANT | NBI | Chlorophyll  (µg cm^‒2^) | Chl b / Chl a | Anth.  (µg cm^‒2^) | Phen.  (µg cm^‒2^) | N  (% DM) | water cont.  (%) |
| --- | --- | --- | --- | --- | --- | --- | --- | --- | --- | --- | --- |
| CRG | C | 29.1 | 3.15 a | 1.79 Ba | 9.7 Ac | 42.2 Ac | 2.24 | 24.07 | 17.1 | 2.3 a | 62.0 B |
|  | ‒UV | 28.3 | 2.04 b | 1.17 Bb | 15.1 Ab | 59.5 Abc | 2.39 | 36 | 17 | 1.7 b | 59.3 B |
|  | +UV | 29.5 | 1.98 b | 1.39 Bb | 16.7 Aa | 75.7 Aa | 2.33 | 53.9 | 17.3 | 2.1 a | 60.5 B |
| CNN | C | 26 | 3.14 a | 1.98 Aa | 9 Bc | 32.45 Bc | 2.17 | 23.4 | 13.9 | 2.2 a | 65.4 A |
|  | ‒UV | 28.6 | 2.13 b | 1.45 Ab | 11.2 Bb | 40 Bbc | 1.58 | 20.1 | 14.7 | 2.4 a | 66.4 A |
|  | +UV | 28.6 | 2.16 ab | 1.48 Ab | 14.3 Ba | 57.82 Ba | 2.18 | 30.2 | 18 | 2.1 a | 65.1 A |
| Sign. | cv | 0.2520 | 0.4540 | 0.0120 | 0.0040 | 0.0050 | 0.734 | 0.8700 | 0.3660 | 0.0370 | 0.0160 |
|  | Treatment | 0.4410 | 0.0001 | 0.0001 | 0.0001 | 0.0001 | 0.974 | 0.1390 | 0.5710 | 0.1950 | 0.9210 |
|  | Interaction | 0.4800 | 0.8270 | 0.5690 | 0.2540 | 0.7400 | 0.950 | 0.4730 | 0.6510 | 0.0000 | 0.7570 |

CHL = (T_850_ ‒T_710_) / (T_710_); where T_850_ and T_710_ are leaf transmittance at 850 and 710 nm; FLA=log (FRF_R_/FRF_UV_); FRF is the far-red Chl fluorescence emission (>710 nm) excited by red (R, 650 nm) and UV (UV, 375 nm) light; ANT = log (FRF_R_ / FRF_UV_); FRF is the far-red Chl fluorescence emission (>710nm) excited by red (R, 650nm) and green (G, 570 nm) light; NBI = [[CHL_AD_ + CHL_AB_) / 2] / [FLA_AD_ + FLA_AB_]; AD and AB represent data from adaxial and abaxial leaf sides, respectively.

**Supplementary Table 3.** Leaf samples fresh, turgid and dry weight (g), dry matter (%) and dry leaf mass per area (Dry LMA), per unit of leaf area (m^‒2^) and per main leaf (leaf^‒1^). Mean values (n=18) and two-way ANOVA significance of differences of cultivar, treatment and interaction effects in season 2018. Different uppercase letters indicate significant differences between cultivar and lowercase letters indicate significant differences among treatments.

| cv | Treatment | Fresh weight  (g) | Turgid weight  (g) | Dry weight  (g) | Dry matter  (%) | Dry LMA  (g m^‒2^) | Dry LMA  (g leaf^‒1^) | RWC  (g g^‒1^) |
| --- | --- | --- | --- | --- | --- | --- | --- | --- |
| CRG | C | 0.421 Ba | 0.480 Ba | 0.158 | 38.0 A | 316.7 | 3.15 Ac | 0.82 A |
|  | ‒UV | 0.376 Bb | 0.424 Bb | 0.148 | 40.7 A | 296.4 | 6.16 Ab | 0.81 A |
|  | +UV | 0.399 Bab | 0.474 Ba | 0.156 | 39.5 A | 312.7 | 7.83 Aa | 0.78 B |
| CNN | C | 0.499 Aa | 0.554 Aa | 0.17 | 34.3 B | 339.7 | 1.60 Bc | 0.86 |
|  | ‒UV | 0.451 Ab | 0.501 Ab | 0.15 | 33.6 B | 300.1 | 1.78 Bb | 0.85 |
|  | +UV | 0.482 Aab | 0.529 Aa | 0.165 | 34.9 B | 329.0 | 1.91 Ba | 0.86 |
| Sign. | cv | 0.0001 | 0.0001 | 0.3250 | 0.0130 | 0.3250 | 0.0001 | 0.013 |
|  | Treatment | 0.0240 | 0.0140 | 0.2170 | 0.8820 | 0.2170 | 0.0001 | 0.774 |
|  | Interaction | 0.9730 | 0.8160 | 0.8580 | 0.7910 | 0.8580 | 0.0001 | 0.677 |

**Supplementary Table 4.** Total anthocyanin and phenolic contents per unit of leaf area (cm^‒2^), measured from flowering until leaf senescence (BBCH 65 – 92). Mean values (n=18) and two-way ANOVA significance of differences of cultivar, treatment and interaction effects in season 2018. Different uppercase letters indicate significant differences between cultivar and lowercase letters indicate significant differences among treatments.

| Pigment | cv | Treatment | BBCH | | | | | |
| --- | --- | --- | --- | --- | --- | --- | --- | --- |
|  |  |  | 65 | 73 | 77 | 83 | 89 | 92 |
| Total anthocyanins  (μg cm^‒2^) | CRG | C | 2.36 b | 1.83 | 4.23 | 33.8 Ab | 47.15 b | 126.8 Aa |
|  |  | ‒UV | 2.02 b | 8.30 | 4.73 | 1.94 Ac | 57.90 b | 64.2 Ab |
|  |  | +UV | 6.20 a | 10.40 | 13.87 | 39.2 Aa | 92.97 a | 153.1 Aa |
|  | CNN | C | 2.99 b | 7.73 | 4.09 | 0.16 Bc | 57.9 b | 67.4 Ba |
|  |  | ‒UV | 1.10 b | 4.76 | 5.57 | 25.96 Ba | 17.8 b | 65.3 Bb |
|  |  | +UV | 3.16 a | 15.70 | 3.6 | 1.89 Bb | 92.97 a | 64.0 Bb |
|  | Sign. | cv | 0.186 | 0.4270 | 0.1430 | 0.0001 | 0.5870 | 0.0001 |
|  |  | Treatment | 0.027 | 0.1040 | 0.1520 | 0.0001 | 0.0210 | 0.0070 |
|  |  | Interaction | 0.158 | 0.4340 | 0.0920 | 0.0010 | 0.6390 | 0.0040 |
| Total phenols  (μg cm^‒2^) | CRG | C | 10.3 Aa | 13.62 Aa | 15.44 A | 18.32 A | 22.01 A | 23.5 |
|  |  | ‒UV | 5.18 Ab | 8.37 Ac | 11.95 A | 16.20 A | 20.78 A | 42.8 |
|  |  | +UV | 6.61 Ab | 11.21 Ab | 15.48 A | 19.92 A | 23.38 A | 27.1 |
|  | CNN | C | 4.95 Bc | 9.11 Bb | 10.91 B | 14.2 B | 19.91 B | 23.7 |
|  |  | ‒UV | 6.73 Bb | 6.89 Bc | 11.93 B | 14.7 B | 17.57 B | 31.5 |
|  |  | +UV | 6.79 Bb | 9.65 Ba | 12.81 B | 15.9 B | 20.19 B | 34.4 |
|  | Sign. | cv | 0.046 | 0.0020 | 0.0050 | 0.0020 | 0.0130 | 0.3060 |
|  |  | Treatment | 0.020 | 0.0050 | 0.0700 | 0.0720 | 0.1980 | 0.2050 |
|  |  | Interaction | 0.008 | 0.0100 | 0.0620 | 0.3490 | 0.3330 | 0.1480 |

**Supplementary Table 5.** Normalized reflectance of leaf samples during season 2018, for: chlorophyll a index, NDCI; chlorophyll b index, ICHL b; carotenoids index, ICAR; TCARI/OSAVI; modified anthocyanin reflectance index, mARI; specific leaf area, ND_LMA_; and dry matter index, NDMI. Mean values (n=60) and two-way ANOVA significance of differences of cultivar, treatment and interaction effects in season 2018. Different uppercase letters indicate significant differences between cultivar and lowercase letters indicate significant differences among treatments.

| DOY |  | CV |  | Treatment | NDCI | TCARI/OSAVI | mARI | ND_LMA_ |
| --- | --- | --- | --- | --- | --- | --- | --- | --- |
| 148 |  | CRG |  | C | 0.34 A | 1.20 Ba | 2.82 B | 0.092 a |
|  |  |  |  | ‒UV | 0.42 A | 0.96 Bb | 3.74 B | 0.084 b |
|  |  |  |  | + UV | 0.42 A | 1.01 Bb | 3.49 B | 0.077 b |
|  |  | CNN |  | C | 0.35 B | 1.23 Aa | 2.52 A | 0.088 a |
|  |  |  |  | ‒UV | 0.32 B | 1.35 Aa | 2.32 A | 0.082 b |
|  |  |  |  | + UV | 0.35 B | 1.30 Aa | 2.54 A | 0.085 b |
|  |  | Sign. |  | CV | 0.001 | 0.0001 | 0.0001 | 0.804 |
|  |  |  |  | Treatment | 0.065 | 0.544 | 0.225 | 0.033 |
|  |  |  |  | Interaction | 0.014 | 0.018 | 0.063 | 0.250 |
| 164 |  | CRG |  | C | 0.48 Aa | 0.81 Bb | 4.07 Aa | 0.108 Ba |
|  |  |  |  | ‒UV | 0.42 Ab | 1.08 Ba | 3.48 Ab | 0.094 Bb |
|  |  |  |  | + UV | 0.47 Aa | 0.83 Bb | 4.06 Aa | 0.085 Bb |
|  |  | CNN |  | C | 0.41 Aa | 1.08 Ab | 3.02 Ba | 0.103 Aa |
|  |  |  |  | ‒UV | 0.36 Ab | 1.31 Aa | 2.33 Bb | 0.099 Ab |
|  |  |  |  | + UV | 0.46 Aa | 0.89 Ac | 2.62 Ba | 0.102 Ab |
|  |  | Sign. |  | CV | 0.029 | 0.009 | 0.001 | 0.055 |
|  |  |  |  | Treatment | 0.007 | 0.011 | 0.014 | 0.005 |
|  |  |  |  | Interaction | 0.343 | 0.364 | 0.461 | 0.012 |
| 184 |  | CRG |  | C | 0.47 b | 0.79 ab | 4.24 c | 0.113 |
|  |  |  |  | ‒UV | 0.50 b | 0.66 ab | 4.94 ab | 0.120 |
|  |  |  |  | + UV | 0.52 a | 0.53 c | 5.34 a | 0.104 |
|  |  | CNN |  | C | 0.51 a | 0.59 c | 5.19 ab | 0.112 |
|  |  |  |  | ‒UV | 0.43 c | 0.96 a | 3.67 c | 0.119 |
|  |  |  |  | + UV | 0.52 ab | 0.59 b | 5.01 b | 0.117 |
|  |  | Sign. |  | CV | 0.363 | 0.422 | 0.462 | 0.304 |
|  |  |  |  | Treatment | 0.009 | 0.011 | 0.060 | 0.092 |
|  |  |  |  | Interaction | 0.006 | 0.012 | 0.013 | 0.231 |

Normalized reflectance indexes formulae: NDCI = (R750 ‒ R705) / (R750 + R705); TCARI/OSAVI = [3 × (R750 ‒ R705) - (0.2 × (R750 ‒ R550) × (R750 / R705))] / [(1 + 0.16) × (R750 ‒ R705) / (R750 + R705 + 0.16)]; mARI = [(1 / 550nm) - (1 / 700nm)] * NIR; ND_LMA_ = R1368 ‒ R1722) / (R1368 + R1722).

**Supplementary Table 6.** Anatomical differences between varieties and among treatments. Thickness of leaf cross-section occupied by palisade and spongy parenchyma, adaxial and abaxial epidermis thickness at veraison (BBCH 83) 2019. Values are mean data (n=6) of cross-section thickness and the percentage of palisade and spongy parenchyma and epidermis, respect to the whole leaf thickness. Different letters indicate significant differences in one-way ANOVA among each combination of cultivar and treatment in season 2019.

| CV | Treatment | Palisade parenchyma | | Spongy parenchyma | | Abaxial + adaxial epidermis (%) | |
| --- | --- | --- | --- | --- | --- | --- | --- |
|  |  | µm | % leaf thickness | µm | % leaf thickness | µm | % leaf thickness |
| Carignano | C | 61.1 a | 32 b | 76.1 b | 40 b | 53 a | 28 a |
|  | +UV | 50.0 b | 38 a | 50.9 d | 38 c | 32 b | 24 b |
|  | ‒UV | 55.3 bc | 38 a | 63.2 c | 43 b | 30 b | 20 c |
| Cannonau | C | 42.3 c | 24 b | 77.3 b | 43 b | 52 a | 33 a |
|  | +UV | 57.1 b | 35 a | 63.6 c | 38 c | 45 a | 27 b |
|  | ‒UV | 45.3 a | 17 c | 162.4 a | 62 a | 55 a | 21 c |
